# Sulphur cycling in a Neoarchaean microbial mat

**DOI:** 10.1111/gbi.12227

**Published:** 2017-01-27

**Authors:** N. R. Meyer, A. L. Zerkle, D. A. Fike

**Affiliations:** ^1^ School of Earth and Environmental SciencesUniversity of St AndrewsSt AndrewsUK; ^2^ Department of Earth and Planetary SciencesWashington UniversitySt. LouisMOUSA; ^3^ Present address: Department of Earth System ScienceStanford UniversityStanfordCA94305USA

## Abstract

Multiple sulphur (S) isotope ratios are powerful proxies to understand the complexity of S biogeochemical cycling through Deep Time. The disappearance of a sulphur mass‐independent fractionation (S‐MIF) signal in rocks <~2.4 Ga has been used to date a dramatic rise in atmospheric oxygen levels. However, intricacies of the S‐cycle before the Great Oxidation Event remain poorly understood. For example, the isotope composition of coeval atmospherically derived sulphur species is still debated. Furthermore, variation in Archaean pyrite δ^34^S values has been widely attributed to microbial sulphate reduction (MSR). While petrographic evidence for Archaean early‐diagenetic pyrite formation is common, textural evidence for the presence and distribution of MSR remains enigmatic. We combined detailed petrographic and in situ, high‐resolution multiple S‐isotope studies (δ^34^S and Δ^33^S) using secondary ion mass spectrometry (SIMS) to document the S‐isotope signatures of exceptionally well‐preserved, pyritised microbialites in shales from the ~2.65‐Ga Lokammona Formation, Ghaap Group, South Africa. The presence of MSR in this Neoarchaean microbial mat is supported by typical biogenic textures including wavy crinkled laminae, and early‐diagenetic pyrite containing <26‰ μm‐scale variations in δ^34^S and Δ^33^S = −0.21 ± 0.65‰ (±1σ). These large variations in δ^34^S values suggest Rayleigh distillation of a limited sulphate pool during high rates of MSR. Furthermore, we identified a second, morphologically distinct pyrite phase that precipitated after lithification, with δ^34^S = 8.36 ± 1.16‰ and Δ^33^S = 5.54 ± 1.53‰ (±1σ). We propose that the S‐MIF signature of this secondary pyrite does not reflect contemporaneous atmospheric processes at the time of deposition; instead, it formed by the influx of later‐stage sulphur‐bearing fluids containing an inherited atmospheric S‐MIF signal and/or from magnetic isotope effects during thermochemical sulphate reduction. These insights highlight the complementary nature of petrography and SIMS studies to resolve multigenerational pyrite formation pathways in the geological record.

## 1 Introduction

The sulphur isotope record has played an integral role in shaping our understanding of key events in Earth's geological and biological history. Surficial S‐cycling principally involves biological and abiotic mass‐dependent fractionation (MDF) processes, which follow a thermodynamically determined, linear δ^33^S/δ^34^S relationship (δ^33^S = 0.515 × δ^34^S). However, the geological S‐isotope record also captures evidence of mass‐independent fractionation (MIF), where δ^33^S and δ^34^S deviate from the predicted terrestrial mass fractionation line, quantified by the capital‐delta (Δ) notation. Prior to the Great Oxidation Event (GOE) at ~2.4 Ga, S‐bearing minerals show large positive and negative Δ^33^S values. After ~2.4 Ga, Δ^33^S ratios diminish towards values that tightly cluster around zero (Δ^33^S = 0 ± 0.2‰). Archaean sulphur MIF has been widely attributed to atmospheric photochemical reactions involving SO_2_; these reactions would have been blocked in the Palaeoproterozoic due to the UV‐shielding effects caused by increased atmospheric O_2_ and O_3_ concentrations (e.g., Farquhar, Bao, & Thiemens, 2000). In addition, the delivery of S‐MIF to the Earth's surface requires sulphur to leave the atmosphere via multiple exit channels at different redox states, which are homogenised when atmospheric oxygen exceeds 10^−5^ of present atmospheric levels (Pavlov & Kasting, 2002). Therefore, measuring multiple sulphur isotopes (δ^34^S, Δ^33^S) in Archaean sediments can provide information on both atmospheric chemistry and biogeochemical sulphur cycling in Archaean palaeoenvironments.

The biogeochemical cycling of sulphur in the Archaean was fundamentally different to the present‐day cycle. The largest flux of sulphur into the modern oceans is riverine sulphate, derived from the oxidative weathering of pyrite. However, this flux was less significant before the GOE due to low atmospheric *p*O_2_; therefore, the two most significant S fluxes into the Archaean oceans were likely hydrothermally sourced and atmospherically derived sulphur species (e.g., Fike, Bradley, & Rose, 2015). The Archaean S‐MIF signal is thought to be dominated by photochemical reactions involving atmospheric SO_2_ (e.g., Farquhar et al., 2000; Pavlov & Kasting, 2002). In contrast, the shielding effect of atmospheric oxygen and ozone prevents the creation of an S‐MIF signal in the contemporary S‐cycle, with the exception of photochemical reactions generating stratospheric sulphate aerosols from volcanic eruptions (Baroni, Thiemens, Delmas, & Savarino, 2007). Furthermore, with low atmospheric and marine *p*O_2_, homogenisation of discrete atmospheric sulphur species (e.g., ${\text{SO}}_{4}^{2-}$, S_*n*_, SO_2_) was limited, further favouring preservation of the Archaean S‐MIF signal.

After formation, the photochemically derived sulphur species, generally assumed to be sulphate (${\text{SO}}_{4}^{2-}$) and elemental sulphur (S_*n*_), were deposited in the oceans. Sulphate is soluble in water and would homogenise rapidly. It could form sulphate‐bearing phases, such as carbonate‐associated sulphate (CAS), gypsum (CaSO_4_.2H_2_O) or barite (BaSO_4_); or it could be reduced to hydrogen sulphide (H_2_S) via microbially mediated MDF redox reactions or thermochemical sulphate reduction (TSR) before capture in the rock record as pyrite. These S‐cycling processes can significantly change the isotope composition of δ^34^S with small (<0.2‰) differential effects on Δ^33^S (Johnston et al., 2005). The fate of S_*n*_ is less certain, but once deposited on the seafloor, it could react with H_2_S produced by microbial sulphate reduction (MSR) to form a reactive, mobile, soluble form of polysulphide $S_{n}^{2-}$. Reactive polysulphide and hydrogen sulphide can then react to form sulphide‐bearing minerals, such as pyrite (Farquhar et al., 2013). Therefore, the S‐isotope composition of pyrite generally records the biogeochemical MDF processes that generated $S_{n}^{2-}$ and H_2_S from atmospheric precursors.

Despite the narrative outlined above, there remain several poorly constrained aspects of the Archaean sulphur cycle. Firstly, the Δ^33^S signatures of the atmospheric products (e.g., S_*n*_ and ${\text{SO}}_{4}^{2-}$) produced through photochemical reactions remain a subject of debate. Archaean rocks from the Hamersley Basin, Western Australia, contain pyrite with S‐isotope ratios that largely follow a linear trend in δ^34^S versus Δ^33^S along an Archaean reference array. Combining these data with photochemical models, Ono et al. (2003) suggested that the Δ^33^S sign was positive for elemental sulphur and negative for sulphate. Furthermore, Palaeoarchaean barites (BaSO_4_) also show a 0 to −1.5‰ Δ^33^S signal (e.g., Roerdink, Mason, Farquhar, & Reimer, 2012; Ueno, Ono, Rumble, & Maruyama, 2008), but these could have formed in the marine water column, in the sediments or in hydrothermal environments not necessarily reflective of the seawater sulphate pool (Paytan, Mearon, Cobb, & Kastner, 2002; Van Kranendonk, 2006). Additional photochemical experiments produce S‐isotope slopes that contrast to Ono et al.'s (2003) results (e.g., see review of Paris, Adkins, Sessions, Webb, & Fischer, 2014); moreover, recent photochemical models demonstrate that the sign in Δ^33^S of experimentally produced sulphur will be highly dependent on the wavelength of UV light used to simulate photolysis (Claire et al., 2014).

Further poorly constrained components of the Archaean S‐cycle are the concentration and δ^34^S of seawater sulphate. Archaean seawater sulphate concentrations were expected to be lower than modern values because of the low atmospheric *p*O_2_ before the GOE. Habicht, Gade, Thamdrup, Berg, and Canfield (2002) used culturing experiments of modern sulphate reducers to observe decreasing fractionation factors with decreasing sulphate concentrations. Assuming this relationship can be extrapolated into the Archaean, they estimated seawater sulphate concentrations at <200 μm. However, MSR fractionation factors are dependent on microbial species (Bradley et al., 2016) and other factors including reduction rate, type of substrate and temperature (e.g., Fike et al., 2009; and references therein). More recent results have suggested Archaean seawater sulphate concentrations could have been even lower, <10 μm (Crowe et al., 2014; Zhelezinskaia, Kaufman, Farquhar, & Cliff, 2014).

The δ^34^S ratio of seawater sulphate depends on the isotope composition of the sulphate input into the oceans, the steady state burial flux of sulphides and sulphate and the fractionation factor between coeval sulphate‐ and sulphide‐bearing species (Fike et al., 2015). Particularly, if the sulphate concentrations in the Archaean oceans were low and basins were restricted, MSR could have changed the residual sulphate composition of seawater through Rayleigh distillative fractionation (Roerdink et al., 2012). Therefore, seawater sulphate S‐isotope compositions may have been heterogeneous, both spatially and temporally, as proposed from the scatter of δ^34^S values preserved in Neoarchaean carbonates (e.g., Paris et al., 2014; Zhelezinskaia et al., 2014).

In addition, the distribution and occurrence of microbial sulphate reduction in the Archaean is debated. Bulk δ^34^S data from Australian Palaeoarchaean barites (Shen & Buick, 2004; Shen, Buick, & Canfield, 2001) along with phylogenetic studies (Wagner, Roger, Flax, Brusseau, & Stahl, 1998) suggest that microbial sulphate reduction is likely an ancient metabolism. However, the link between MSR S‐isotope fingerprints and pyrite textures remains enigmatic. Ono, Beukes, and Rumble (2009) sampled multiple pyrite phases in ~2.5‐Ga upper Prieska facies from the GKP01 Agouron drill core. They correlated different pyrite phases with the Archaean reference array (Ono et al., 2003) and suggested nodular and layered pyrite had S‐isotope signatures most consistent with MSR. Similarly matching pyrite crystal morphology to the array, Kamber and Whitehouse (2007) proposed spheroidal pyrite concretions capture an MSR S‐isotope fingerprint in the 2.52‐Ga Upper Campbellrand Subgroup, Transvaal, South Africa. Moreover, Fischer et al. (2014) showed that δ^34^S in nodular pyrite were characterised by systematic enrichment towards the rims (with the lowest δ^34^S signature present in the centre of the nodules). Additional studies in the Griqualand West Basin are required to test whether these textural and geochemical interpretations can be applied to pyrite from other palaeoenvironments and depositional ages.

To address these gaps in our current understanding of Archaean sulphur cycling, we measured in situ, μm‐scale, multiple sulphur isotope ratios (δ^34^S and Δ^33^S) in exceptionally well‐preserved pyritised microbialites in shales (sample‐3184) from the ~2.65‐Ga Lokammona Formation, Ghaap Group, South Africa. By combining petrography with S‐isotope data from secondary ion mass spectrometry (SIMS), we aim to increase our understanding of sulphur cycling in a Neoarchaean microbial mat, and the secondary (late‐diagenetic/post‐lithification) processes that may impact the primary (depositional/early‐diagenetic) S‐isotope signal. By petrographically correlating pyrite phases with sulphur isotope fingerprints, we can determine the MDF and MIF processes that contributed to the formation of multiple pyrite generations in these sediments.

## 2 Materials and Methods

### 2.1 Geological setting and core material

**Figure 1:**
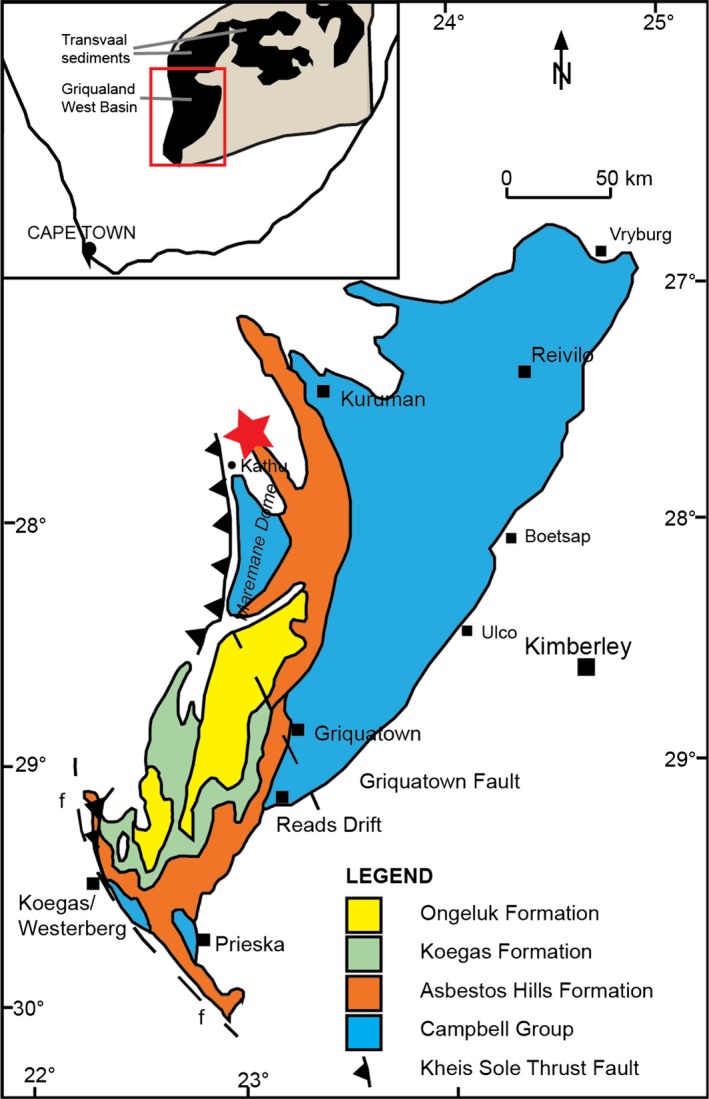
Simplified geological map of Transvaal and Griqualand West sediments, the approximate location of BH1‐SACHA (star) and the inferred fault trace of the Kheis sole thrust fault. Insert: the red rectangle shows the location of the main map relative to other Transvaal sediments and the Kaapvaal craton (beige). Adapted from Altermann and Wotherspoon (1995)

**Figure 2:**
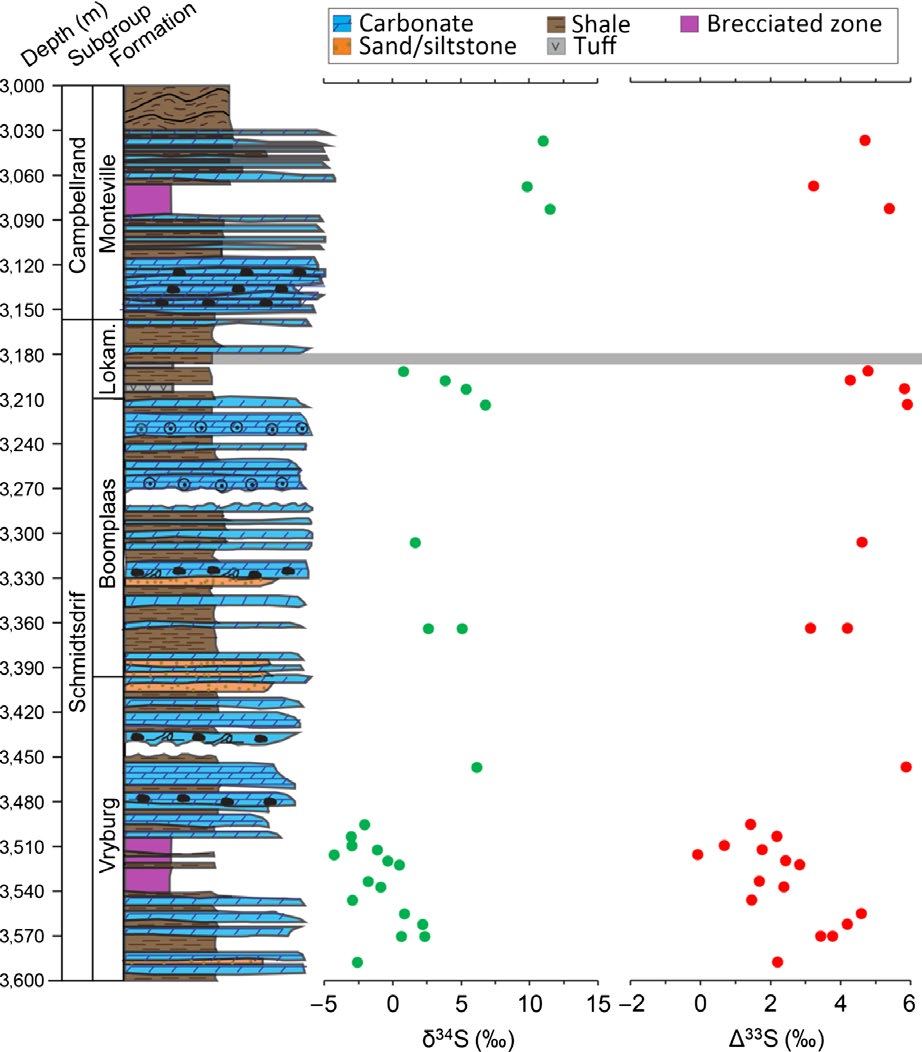
BH1‐SACHA bulk sulphur isotope data plotted against core depth (m) with the corresponding sedimentary log. Brecciated zones largely reflect post‐depositional tectonic deformation. The pyritised microbialites analysed in this study were sampled at a depth of 3,184 m (grey band). Lokam., represents Lokammona. Data from Izon et al. (2015) and stratigraphic log adapted from Altermann & Siegfried, 1997

Our sample (3184) was collected from the BH1‐SACHA core, drilled through the Neoarchaean Transvaal Supergroup in the Griqualand West Basin (Figure 1). The core material was obtained from the National Core Library at Donkerhoek (Pretoria, South Africa). The core contains carbonates, siliciclastics and iron formations as well as several igneous intrusive bodies (Figure 2). Sample‐3184 was obtained from a depth of 3184 m from the Lokammona Formation, Schmidtsdrif Subgroup, Ghaap group, Transvaal Supergroup. The Lokammona (Clearwater) Formation is dominated by shale, tuff layers intercalated with black shale, and minor dolomites (Figure 2). The high proportion of fine‐grained sediments is consistent with deposition in a low‐energy environment; Altermann and Siegfried (1997) suggested sedimentation occurred in a deep, shelf environment with shale‐carbonate/shale cycles representing shallowing‐upward cycles. They also noted the presence of microbial laminites, which suggested deposition in the photic zone. The interpretation is supported by studies suggesting that modern microbial mats that produce microbially induced sedimentary structures (MISS) are dominated by benthic photoautotrophs (Noffke, 2009). The Lokammona Formation has a model age of 2.650 ± 0.008 Ga from SHRIMP U‐Pb analyses of zircons from tuff laminae (Knoll & Beukes, 2009).

The area sampled by the BH1‐SACHA core is characterised by little subsequent tectonic deformation (Beukes, 1987), and it has been subjected to sub‐greenschist facies metamorphism (Button, 1973; Miyano & Beukes, 1984). The core has been penetrated by dykes and sills; the largest continuous igneous intrusion occurs at a depth of 922.3–1201.27 m. In the Lokammona Formation, there is some evidence of secondary mineralisation, particularly at ~3,165 m where galena has been identified (Altermann & Siegfried, 1997). We will consider these igneous and metasomatic processes when interpreting geochemical data from BH1‐SACHA.

### 2.2 Imaging and EPMA

We used the VHX‐2000 super‐resolution digital microscope housed in the School of Earth and Environmental Sciences at the University of St Andrews (Scotland, UK) to image thick sections of sample‐3184. This facilitated the detailed mapping of structures and fabrics within the sample and selection of target areas for SIMS. Backscatter electron (BSE) imaging was carried out using the School of Earth and Environmental Sciences’ Jeol JCXA‐733 Superprobe electron microprobe analyser (EPMA). Prior to analysis, sample‐3184 thick sections were coated with a ~40‐nm‐thick graphite layer. The analysis was performed using a primary ion current of ~17–21 nA and an acceleration voltage of 15 kV.

### 2.3 Multiple sulphur isotope analyses via SIMS

The sulphur isotope data are reported relative to the Vienna Canyon Diablo Troilite (V‐CDT) international reference standard. The delta (δ) notation denotes the deviation of the sample from V‐CDT in permil (‰; Equations (1), (2)). The capital‐delta (Δ) reflects mass‐independent fraction, quantifying the deviation of a sample from the expected terrestrial mass fractionation line (Equation (3)).

(1) $$\delta^{33}S=\left(\frac{{{(}^{33}S{/}^{32}S)}_{\mathrm{sample}}}{{{(}^{33}S{/}^{32}S)}_{V-\mathrm{CDT}}}-1\right)\times 1000$$

(2) $$\delta^{34}S=\left(\frac{{{(}^{34}S{/}^{32}S)}_{\mathrm{sample}}}{{{(}^{34}S{/}^{32}S)}_{V-\mathrm{CDT}}}-1\right)\times 1000$$

(3) $$\Delta^{33}S=\delta^{33}S_{V-\mathrm{CDT}}-1000\times \left(1-\left(\frac{\delta^{34}S_{V-\mathrm{CDT}}}{1000}\right)0.515\right)$$

Multiple sulphur isotope analyses (δ^34^S and δ^33^S) were conducted using the CAMECA IMS 7f‐GEO secondary ion mass spectrometer housed in the Department of Earth and Planetary Sciences at Washington University (St Louis, MO, USA). Secondary ion mass spectrometry (SIMS) enables the in situ measurement of S‐isotope ratios to a high spatial resolution, precision and mass resolution while minimising destruction to the sample. Epoxy thick sections of sample‐3184 with a 2.54 cm diameter were made, polished to <1 μm surface roughness and covered with a ~50‐nm gold coating.

Samples were outgassed in an ancillary chamber at <1 × 10^−8^ mB prior to analysis, and the pressure in the sample chamber was allowed to equilibrate for >1 hr after sample introduction, prior to analysis. Thick sections were analysed in a vacuum with a pressure of 3 × 10^−9^ mB. A focused ~2 μm Cs^+^ primary ion beam of 0.9–1.8 nA was rastered over a 10 μm by 10 μm area of interest. Each measurement was divided into 15 cycles and required 7–8 min to complete. The secondary ions were collected using faraday cups (FC). The 7f‐GEO is specifically designed for precise isotope ratio measurement using two FCs optimised for major (FC1) and minor (FC2) isotope data acquisition in “charge mode” (Peres, de Chambost, & Schuhmacher, 2008). Each isotope is selected by magnet switching, and the corresponding secondary ion counts were collected in sequence of ascending mass. The desired FC is selected using a deflector situated in the detector assembly. This configuration allows for the initiation of data acquisition from FC2 prior to complete dissipation of the signal from FC1. This alternating collection arrangement results in improved signal‐to‐noise ratios and thereby greater precision per unit time. In this study, two minor isotopes were to be analysed. As back‐to‐back data collection using FC2 would defeat the signal‐to‐noise advantage described above; thus, a pseudomeasurement, at mass 33.5, was made on FC1 so that each cycle became ^32^S (FC1), ^33^S(FC2), 33.5(FC1), ^34^S(FC2). The background level for FC1 was ~3 × 10^4^ counts/s and for FC2 ~3 × 10^3^ counts/s. Typically, secondary ion yields were: ^32^S~2 × 10^8^ counts/s, ^33^S~10^6^ counts/s and ^34^S~10^6^ counts/s.

Background and ion yields were recalibrated on a daily basis. Mass calibration, as well as automatic peak centring of the field and contrast apertures, was performed at the start of each measurement; this corrects for any drift in the secondary ion beam or the magnetic field (Fike et al., 2009). To remove the 50‐nm gold coating, each measurement was pre‐sputtered for 2 min prior to the start of the 15 cycles. A minimum mass resolving power (MRP) of 3900 is required to separate the ^33^S and ^32^SH peaks, and therefore, a MRP of ~4300 was used. Analysis was preferentially carried out in regular spaced intervals in a grid‐like pattern (Fike, Gammon, Ziebis, & Orphan, 2008). Generally, the analysis spots were placed at ~50‐μm intervals. However, deviations from the ideal grid were necessary to consistently sample pyrite instead of siliciclastic matrix.

The internal error (standard error of *n* = 15 cycles) varied inversely with the ^32^S count rate (Figure S1), suggesting that precision was limited by counting statistics (Fike et al., 2009). Anomalous data were omitted from further analysis if any of the following applied to the measurement:

1. The sputter area clearly targeted only matrix as subsequently determined from BSE and super‐resolution digital microscope images.
2. The uncorrected relative standard error for ^32^S counts was >1%.
3. The uncorrected relative standard error for ^34^S/^32^S was >0.1%.

After excluding the anomalous measurements, the internal error was typically <1‰ for δ^34^S and <0.5‰ for δ^33^S (1*SE*). An in‐house Washington University pyrite standard was mounted in a polished and gold‐coated thin section and placed in a separate holder. Its composition was determined by measuring the S‐isotope composition of the internal standard 12 times and the Balmat standard 20 times. The Balmat standard (δ^34^S = 15.1‰ and δ^33^S = 7.7‰) analyses bracketed the in‐house standard. The in‐house standard has a known isotope composition of δ^34^S = 0.13 ± 0.30‰ and δ^33^S = 0.13 ± 0.20‰ (1*SE*). The external errors (1 standard deviation of multiple adjacent points on the in‐house standard) were typically 0.38‰ and 0.32‰ for δ^34^S and δ^33^S, respectively (*n* = 35). Within sample‐3184, there were 26‰ variability in measured δ^34^S values and 14‰ variability in δ^33^S. This variation far exceeds the calculated internal and external errors.

On average, a session consisted of ~50 measurements of unknowns and was bracketed by ≥4 measurements of the in‐house standard on both sides of the unknowns (Figure S2). Instrumental mass fractionation (IMF) was corrected for by standard‐sample bracketing. The mean, uncorrected isotope composition of repeated analyses of the in‐house pyrite standard on the SIMS was δ^34^S = −0.05 ± 0.38‰ and δ^33^S = 1.00 ± 0.32‰ (1σ; *n* = 35; relative to V‐CDT). Therefore, the IMF (IMF = R_raw_/R_known_) was typically 1.000 and 1.001 for ^34^R and ^33^R, respectively. Although IMF can vary according to instrumental conditions, the S‐isotope offset between bracketing standards and unknowns should not vary. Furthermore, the measured S‐isotope ratios of a session's bracketing standards were observed to determine drift. The drift value was usually <1‰ over 24 hr. To correct for this, a linear and constant drift was assumed within a session. The magnitude of this drift was much smaller than the signals observed and was therefore unlikely to have impacted the results.

## 3 Results

### 3.1 Textural analysis

**Figure 3:**
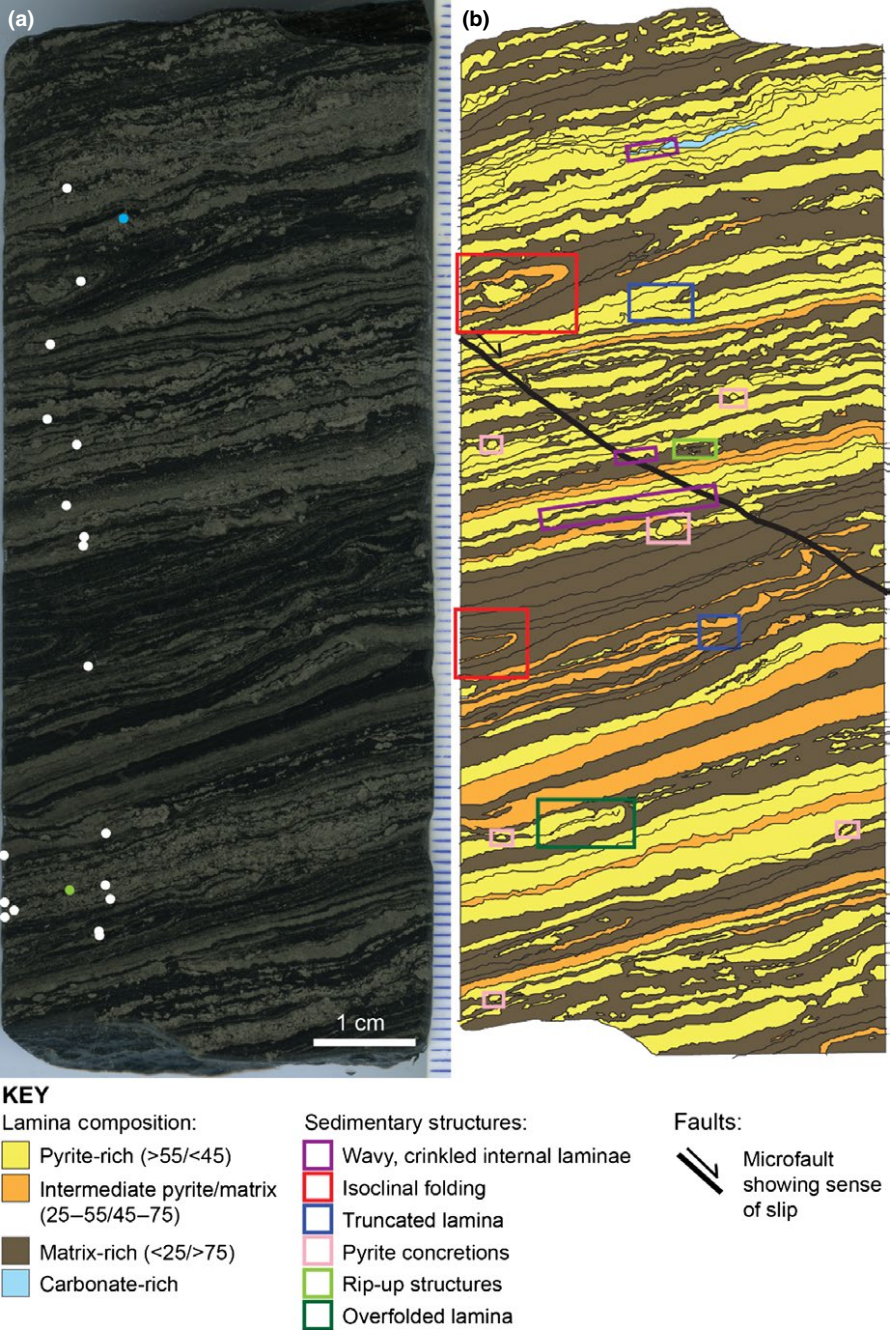
(a) Reflected light scanner image of sample‐3184 from the Lokammona Formation showing the areas of SIMS analyses (white dots). Blue dot = Figure 6 analysis area; green dot = Figure 7 analysis area. Divisions on the scale bar represent 1 mm. (b) A trace of the image in (a) showing laminae compositions, type examples of sedimentary structures discussed in the text and the location of the normal microfault. Modal percentages of laminae compositions are shown in brackets (pyrite/matrix)

**Figure 4:**
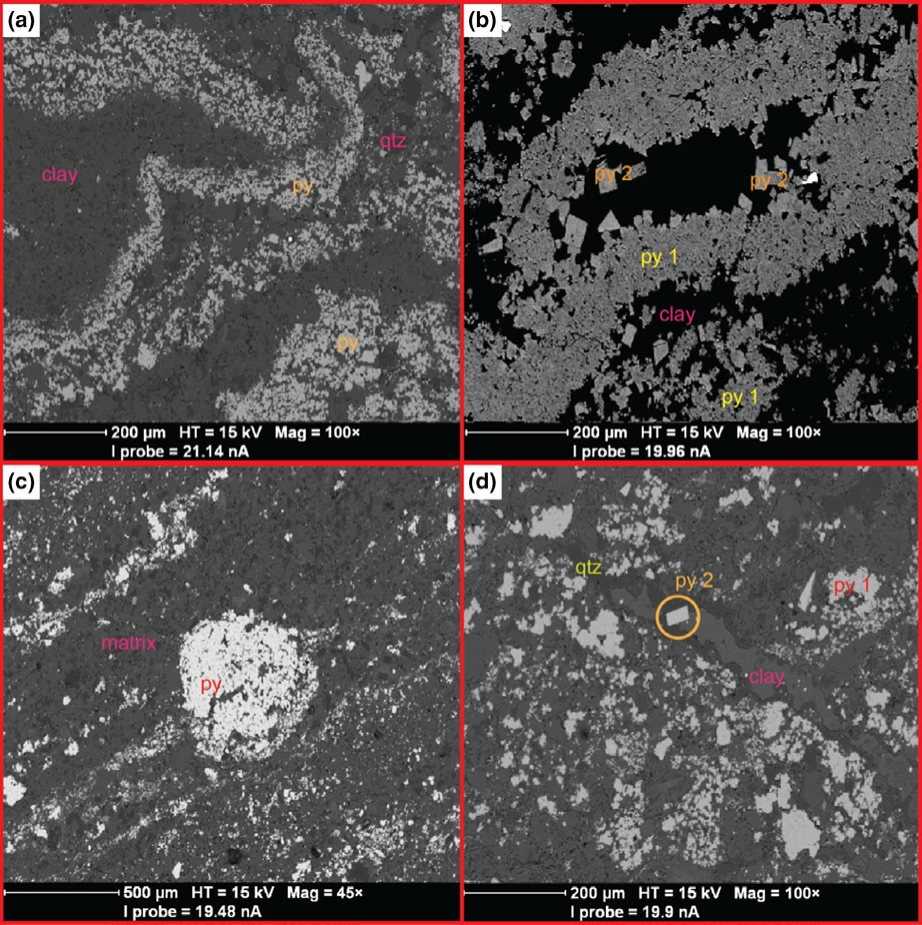
BSE images of different sedimentary structures in sample‐3184. (a) Wavy crinkled internal lamination composed of type 1 pyrite and disseminated pyrite. (b) Wavy crinkled internal lamination composed of type 1 pyrite with type 2 pyrite overgrowths. (c) Pyrite concretion. Note that the concretion both cross‐cuts and causes deformation of the lamination. (d) The normal microfault that is infilled by quartz and clay minerals. Note the microfault and vein cross‐cut type 1 pyrite; type 2 pyrite cross‐cuts the microfault and vein. Therefore, the relative order of formation is as follows: pyrite 1 precipitated first, brittle deformation caused microfault formation, the microfault was infilled, and finally type 2 pyrite precipitated. py, py 1, py 2 and qtz represent pyrite, type 1 pyrite, type 2 pyrite and quartz, respectively

Sample‐3184 is a black shale composed of ~1‐mm‐thick laminae with varying proportions of pyrite and matrix (Figure 3). The matrix is composed of silt‐sized particles (~40 μm) of aluminosilicates (clay minerals; 80% modal abundance) and quartz (20%). Uncommon <5‐μm detrital grains of rutile (TiO_2_) and apatite (Ca_10_(PO_4_)_6_(F,Cl,OH)_2_) make up <1% of the matrix volume. Laminae contain variable proportions of pyrite‐to‐matrix ratios, ranging from 10%–70% pyrite to 30%–90% matrix. There are three distinguishable pyrite phases: A) disseminated pyrite crystals, <5 μm in size; B) *type 1 pyrite*: irregular aggregates of coalesced pyrite crystals. Clusters can range from 10 μm to 500 μm across, and are composed of anhedral to subhedral, 1‐ to 20‐μm pyrite crystals. (Figure 4); C) *type 2 pyrite*: euhedral cubic pyrite with a crystal size ranging from 10 to 100 μm (Figure 4). Some euhedral pyrite crystals are isolated within the matrix, while other crystals overgrow and encrust type 1 aggregates. Overgrowths occur on the edges of type 1 pyrite aggregates and within clusters where matrix lenses occur. Type 1 and type 2 pyrites are chemically distinct phases, as determined from BSE images (Figures 4 and S6).

Laminae containing type 1 pyrite show sedimentary structures that suggest it precipitated during early diagenesis, prior to soft sediment deformation. These include wavy crinkled laminae with typical wavelengths of ~500 μm and wave heights of ~200 μm, isoclinal folds, 1‐mm rip‐up structures and over‐folded laminae. Particularly, <2‐mm irregular, ellipsoidal concretions are composed of polycrystalline type 1 pyrite. They cross‐cut lamination but also cause draping of laminae on either side of the concretion (Figures 4 and S4); this suggests type 1 pyrite precipitated after deposition but before lithification, during early diagenesis.

A normal microfault cross‐cuts the entire core section of sample‐3184 (Figure 3). The ~50‐μm‐wide microfault has been infilled by aluminosilicates and silica. The microfault cross‐cuts type 1 pyrite; type 2 pyrite crystals cross‐cut the microfault itself (Figure 4). Furthermore, type 2 pyrite overgrows the sedimentary structures composed of type 1 pyrite. According to these cross‐cutting relationships, type 2 pyrite precipitated post‐lithification.

### 3.2 Multiple sulphur isotope data

**Figure 5:**
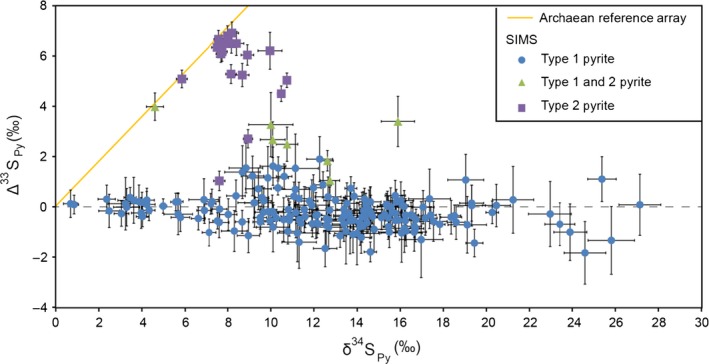
Plot of multiple S‐isotope data (δ^34^S and Δ^33^S) measured via SIMS. SIMS error bars are 1*SE* for each measurement (*n* = 15 cycles). Green triangles represent a mixed signal, where the area of analysis sampled both type 1 and type 2 pyrite. The orange line is the *Archaean reference array* as described by Ono et al. (2003)

**Figure 6:**
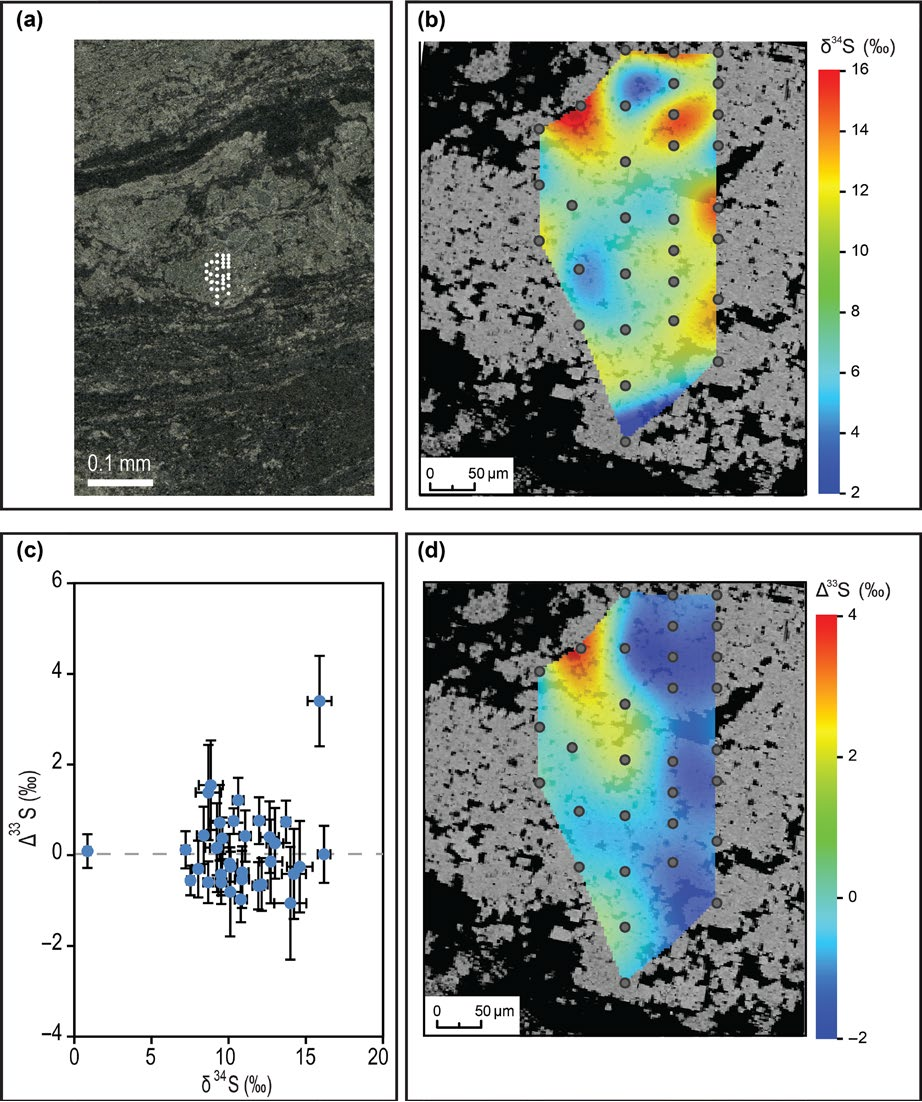
Combined petrography with SIMS S‐isotope data shows the typical textural, δ^34^S and Δ^33^S characteristics of type 1 pyrite. (a) Reflected light photomicrograph of the analytical grid location. (b) BSE image of the analysis area overlain by a SIMS δ^34^S image constructed by spline interpolation of the analytical grid (*n* = 31). Note that the analysis area is composed of a ~500‐μm aggregate of anhedral to subhedral, 1‐ to 20‐μm pyrite crystals. (c) Plot to show SIMS δ^34^S against Δ^33^S data (‰). Note the variation in δ^34^S data is significant; the variation in Δ^33^S data is not significant. Error bars represent 1*SE* for each measurement (*n* = 15 cycles) (d) BSE image of the analysis area overlain by a SIMS Δ^33^S image constructed by spline interpolation of the analytical grid (*n* = 31)

**Figure 7:**
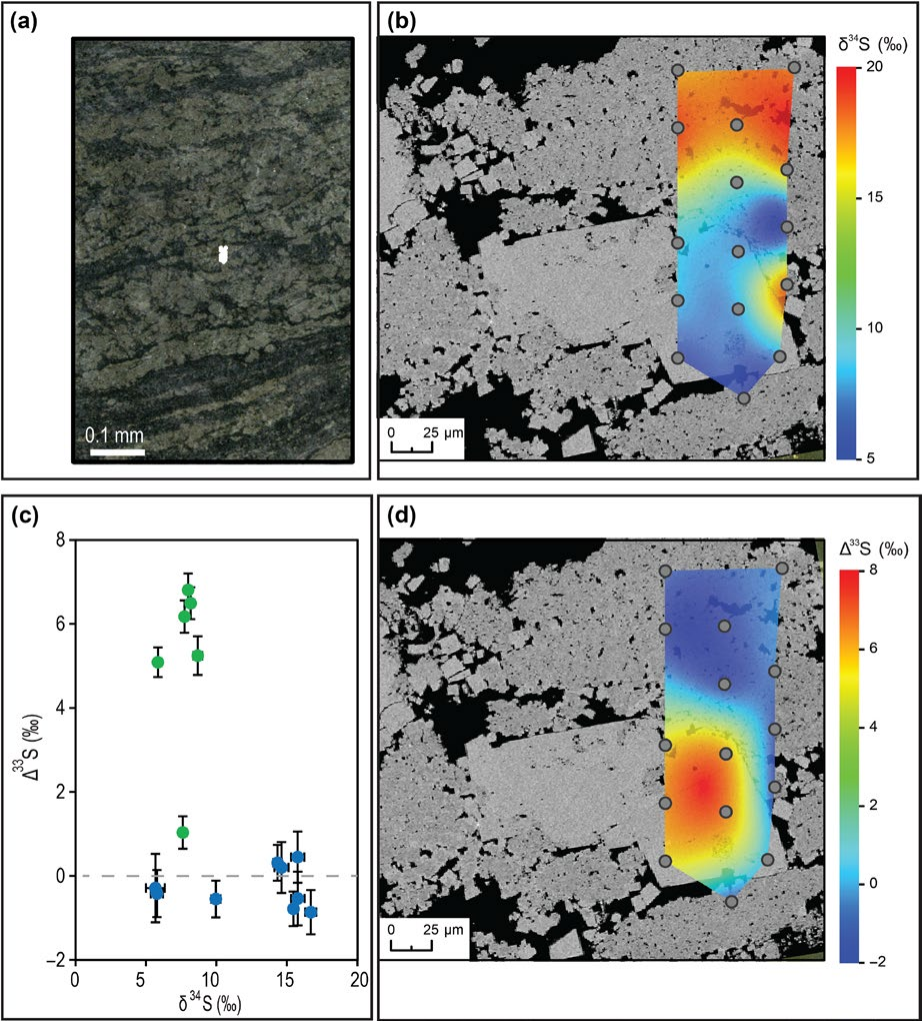
The contrasting textural and S‐isotope signatures of type 1 and type 2 pyrite. (a) Reflected light photomicrograph of the area of analysis. (b) BSE image of the analysis area overlain by a SIMS δ^34^S image constructed by spline interpolation of the analytical grid (*n* = 15). Note the ~300‐μm cubic type 2 pyrite grain, overgrowing type 1 pyrite aggregates. (c) Plot to show SIMS δ^34^S against Δ^33^S data (‰). Note the significant difference in Δ^33^S for type 1 and 2 pyrite. Error bars represent 1*SE* for each measurement (*n* = 15 cycles). Blue circles = type 1 pyrite; green circles = type 2 pyrite. (d) BSE image of the analysis area overlain by a SIMS Δ^33^S image constructed by spline interpolation of the analytical grid (*n* = 15)

All the SIMS data measured in sample‐3184 are presented in Figure 5. There is no obvious stratigraphic trend; however, there is a relationship between pyrite crystal shape and S‐isotope compositions. The first data set shows a mean of δ^34^S = 12.54 ± 4.98‰ and Δ^33^S = −0.21 ± 0.65‰ (±1σ, *n* = 177) and corresponds to type 1 pyrite (Figures 6 and 7). In sample‐3184, type 1 pyrite δ^34^S values vary by 26‰; within a ~500 × 500 μm area, the range of δ^34^S is 15‰ (Figure 6). Wavy crinkled laminae show δ^34^S and Δ^33^S values consistent with typical type 1 pyrite (Figure S3). The second data set has a mean of δ^34^S = 8.36 ± 1.16‰ and Δ^33^S = 5.54 ± 1.53‰ (±1σ, *n* = 18) and was measured in type 2 pyrite (Figures 7 and S5). To a 95% confidence level, the S‐isotope values for type 1 and type 2 pyrite are significantly different in their medians and variances (*p* = .000 for δ^34^S and Δ^33^S in two‐sample Wilcoxon and Levene's tests). Some data points correspond to an intermediary S‐isotope signature between the type 1 and type 2 pyrite end members. When these measurements were subsequently examined in their petrographic contexts, photomicrographs suggest the analysis spots targeted a mixture of both pyrite phases.

## 4 Discussion

### 4.1 Early‐diagenetic (type 1) pyrite

Laminae composed of type 1 pyrite are wavy and crinkled on a sub‐mm scale (Figures 3 and 4) and are consistent with sedimentary structures that have been microbially induced (Noffke, 2009). Prokaryotes and eukaryotes in microbial mats produce a matrix of extracellular polymeric substances (EPS), which are composed of polysaccharides, proteins, humic substances and nucleic acids (Nielsen, Jahn, & Palmgren, 1997). The matrix has several physical functions, including adhesion to surfaces, aggregation of cells, stabilisation of microbial mat structure, sorption of exogenous organic molecules and retention of water (Laspidou & Rittmann, 2002). Importantly, EPS is responsible for the formation of wavy crinkled lamination due to its ability to trap detrital grains and its high cohesive properties. This cohesion is also responsible for the formation of over‐folded layers (Figure 3), which occur when the mat surface is eroded and turned over at its edges (Schieber, 1999). However, wavy crinkled laminae can also form through the differential compaction of phyllosilicates around, for example, microconcretions, silt lenses or silica spherules (Schieber, 2007). Examples of compaction around such structures were absent in BSE images of sample‐3184 (Figure 4). Thus, the sedimentary structures indicate high cohesion of the depositional layers and are consistent with typical microbialite textures, supporting a biogenic control on the formation of type 1 pyrite.

The S‐isotope composition of type 1 pyrite is distinguishable by a Δ^33^S value of −0.21 ± 0.65‰ (±1σ) and a 26‰ range in δ^34^S within sample‐3184. The Δ^33^S error bars for type 1 pyrite within an area of analysis overlap (Figure ;6); therefore, the Δ^33^S variation within a lamina is statistically insignificant. The considerable variation in δ^34^S values is consistent with MSR—modern microbial mats with sulphate reducers show ~15–53‰ variations in δ^34^S on a 1‐mm scale (Fike et al., 2009; Wilbanks et al., 2014), similar to what we observe here. Thus, both textural evidence and the S‐isotope composition are consistent with the expected biosignatures of an ancient microbial mat containing sulphate reducers. The 26‰ range in δ^34^S within sample‐3184 could be explained by non‐uniformity in MSR fractionation factors, which depend on the microbe strain, type of electron donors, reduction rate, temperature and the presence of additional S metabolisms such as oxidation and disproportionation (e.g., Fike et al., 2009).

**Figure 8:**
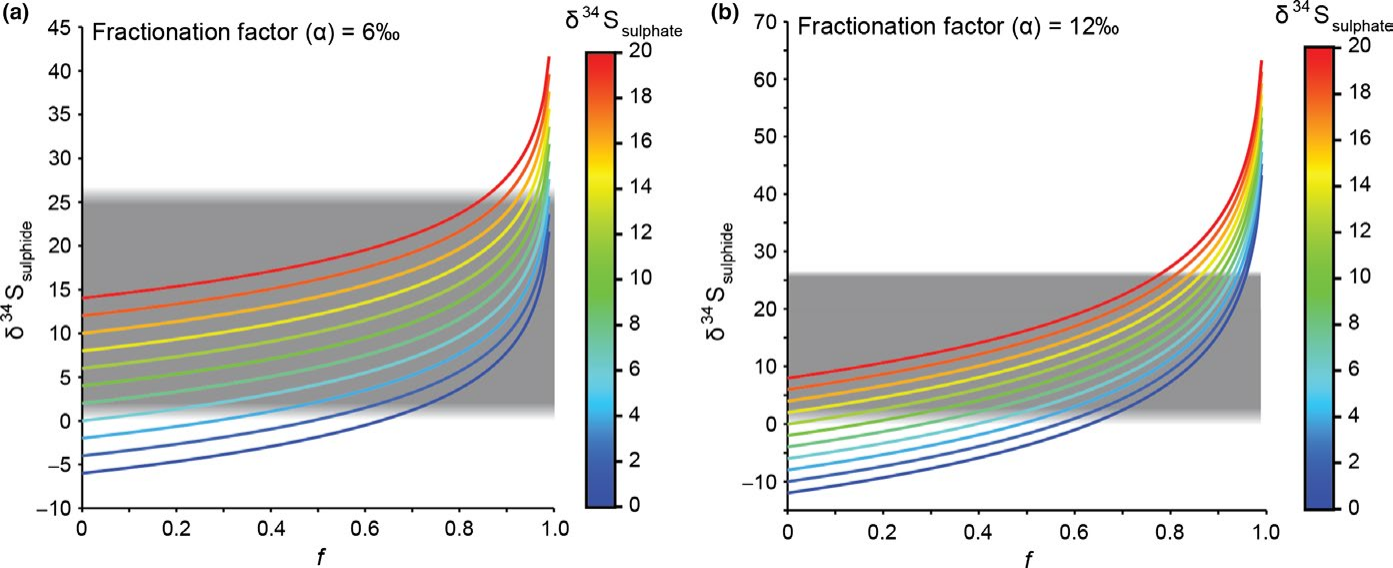
Graphs showing the calculated δ^34^S values of the hydrogen sulphide instantaneous product relative to the proportion of sulphate consumed (*f*), the initial δ^34^S_sulphate_ composition and the MSR fractionation factor (α_source‐product_). The grey rectangles correspond to the range of δ^34^S_sulphide_ compositions measured in type 1 pyrite in sample‐3184. (a) α = 6‰, (b) α = 12‰

Alternatively, the scatter and enrichment in δ^34^S sulphide relative to coeval sulphate could be produced by fractionation during MSR in a (partially) closed system, analogous to a mat environment periodically flushed with seawater sulphate. We used a Rayleigh distillation model to explore this scenario, using a suite of realistic fractionation factors for MSR (α_MSR_ = 1,000 × [^34^R_sulphate_/^34^R_sulphide_ − 1], where ^34^R = ^34^S/^32^S) and variable seawater δ^34^S_sulphate_ values. Figure 5 shows that few data occur above 18‰, and thus, the curve showing the δ^34^S_sulphide_ of the instantaneous product must be in the distillative tail when δ^34^S_sulphide_ > 18‰. Values of 6‰–12‰ for α_MSR_, 4‰–16‰ for δ^34^S_sulphate_ and *f* values from ~0.5 to 0.9 are all consistent with the data (Figure 8).

This 6‰–12‰ estimate of α_MSR_ is at the lower end of the spectrum for the modern MSR cell‐specific fractionation factors of α_MSR_ = 2‰–66‰ (Fike et al., 2015). Small MSR isotope fractionation can occur when sulphate concentrations are low, when sulphate reduction rates or sulphide oxidation rates are high, when H_2_ is used as an electron donor instead of organic carbon, and/or when sulphur disproportionation levels are low (e.g., Fike et al., 2009 and references therein). Although the relationship between sulphate concentration and MSR fractionation factor is complex (see Bradley et al., 2016), our estimates of α_MSR_ are consistent with other studies that suggest low seawater sulphate concentrations and high rates of MSR in the Archaean (e.g., Crowe et al., 2014; Habicht et al., 2002; Zhelezinskaia et al., 2014). Furthermore, the seawater δ^34^S compositions we estimate from closed‐system modelling of the SIMS data are consistent with estimates of seawater sulphate δ^34^S from Neoarchaean CAS (e.g., Domagal‐Goldman, Kasting, Johnston, & Farquhar, 2008; Guo et al., 2009; Paris et al., 2014). However, the small MSR fractionation factors and variation in Δ^33^S values in the literature imply Archaean seawater sulphate had a short residence time due to a small reservoir size relative to the fluxes into and out of the reservoir; thus, the S‐isotope composition of seawater sulphate was likely spatially and temporally heterogeneous (Fischer et al., 2014; Zhelezinskaia et al., 2014).

We therefore conclude that type 1 pyrite reflects the morphological and geochemical signatures of sulphate reducers in a Neoarchaean microbial mat, as inferred from (i) the biogenicity of sedimentary structures like wavy crinkled lamination, (ii) textural evidence of early‐diagenetic precipitation of type 1 pyrite, and (iii) models of type 1 pyrite δ^34^S values supporting plausible MSR fractionation factors expressed within a restricted seawater sulphate pool.

### 4.2 Secondary (type 2) pyrite

As discussed above, simple cross‐cutting relations suggest type 2 pyrite precipitated after type 1 pyrite formation and post‐lithification, because it overgrows deformed aggregates of type 1 pyrite, and it cross‐cuts a normal microfault with vein infill (Figure 4). The low standard deviation of type 2 pyrite S‐isotope ratios suggests these crystals have all been formed from the same, uniform S‐isotope source. The δ^34^S and Δ^33^S signature of type 2 pyrite falls within Ono et al.'s (2003) estimates of the composition of atmospheric elemental sulphur as inferred from Archaean pyrite data and photochemical experiments (Figure 5), and Paris et al.'s (2014) Neoarchaean CAS values. Similar δ^34^S and Δ^33^S ratios in euhedral grains sampled from GKF‐01 (a core sampling deeper water equivalents of the BH1‐SACHA; Schröder, Lacassie, & Beukes, 2006) were interpreted as having an atmospheric elemental sulphur origin (Farquhar et al., 2013). Farquhar et al. (2013) hypothesised that solid atmospherically derived S_*n*_ particles could remain in an unreactive form as they fell through the water column. After deposition, they could then react with H_2_S in the sediment, producing reactive polysulphide. Finally, the polysulphide could react with FeS to form pyrite. Farquhar et al. (2013) predicted that the pyrite would closely reflect the S‐isotope signature of atmospheric elemental sulphur if the mass contribution of H_2_S was small relative to polysulphide in the pyrite product.

A similar interpretation of type 2 pyrite formation in sample‐3184 is less likely, due to its inferred later timing of formation based on the petrographic relationships we describe above. As type 1 pyrite likely formed from sulphide generated via microbial sulphate reduction, H_2_S was abundant during early diagenesis. Therefore, any atmospheric, unreactive elemental sulphur particles that deposited into the Neoarchaean microbial mat would have simultaneously reacted with H_2_S to form a soluble, reactive form of polysulphide. During early diagenesis, the mobile polysulphide, a precursor to pyrite synthesis, would have reacted with an iron monosulphide to form FeS_2_. However, petrographic relationships suggest type 2 pyrite formed post‐lithification, not coevally with type 1 pyrite during diagenesis (Figure 4). Therefore, type 2 pyrite in sample‐3184 unlikely formed from an elemental sulphur source by the mechanism proposed by Farquhar et al. (2013). Hence, we suggest the Δ^33^S = 5.5‰ signature of type 2 pyrite was not produced from contemporaneous atmospheric S_*n*_ products at the time sample‐3184 was formed. Instead, we propose later‐stage alteration processes could explain the Δ^33^S values measured in type 2 pyrite.

The BH1‐SACHA core has been altered by several post‐depositional hydrothermal events that could have caused multigenerational pyrite genesis. Stratigraphic logs indicate evidence of secondary mineralisation of galena, ~20 m stratigraphically higher than sample‐3184. Furthermore, intrusion of the igneous body at 922.3–1201.27 m formed a contact metamorphic aureole (Altermann & Siegfried, 1997). To the best of our knowledge, this dyke has not been dated; however, it may be co‐genetic to other mafic intrusions related to the emplacement of the Bushveld complex, 2.06–2.05 Ga (Hartzer, 1995). Associated igneous and metamorphic fluids can catalyse metasomatic reactions, causing the loss and gain of elements in circulating fluids. The occurrence of metasomatism in BH1‐SACHA is supported by regional geological evidence; a platform‐wide fluid flow event at ~2.0 Ga could have caused the Pb‐Zn‐Cu‐mineralisation in the Campbellrand Subgroup, Ghaap Group. Huizenga, Gutzmer, Greyling, and Schaefer (2006) sampled Mississippi Valley‐type (MVT) deposits in the Griqualand West area, ~100–120 km straight‐line distance from Kathu. They estimated a regional fluid temperature of 200–240°C and pressure of 0.8–1.5 kbar during mineralisation of the MVT deposits. It is possible that BH1‐SACHA experienced similar metamorphic conditions. However, Altermann and Siegfried (1997) noted evidence of thrust faults in BH1‐SACHA at depths >2,800 m in the Monteville Formation. They suggested this is the intersection of BH1‐SACHA with the Kheis sole thrust fault (Figure 1). Allochthonous sections of Archaean Transvaal Supergroup rocks (including rocks that were sampled by the BH1‐SACHA core) as well as Proterozoic Waterberg and Olifantshoek Groups were horizontally displaced and now lie unconformably on the autochthonous Archaean package of rocks (Altermann & Wotherspoon, 1995; Martini, Eriksson, & Snyman, 1995). The age assigned to thrusting ranges from 2.20 to 1.75 Ga (Grobbelaar, Burger, Pretorius, Marais, & Van Niekerk, 1995). If the thrust is older than ~2.0 Ga, it suggests that the hanging wall rocks were exhumed before the key igneous and metasomatic events associated with the Bushveld Complex. Hence, the metamorphic grade of BH1‐SACHA in the hanging wall may be lower than the footwall MVT deposits examined by Huizenga et al. (2006). In addition, De Kock et al. (2009) showed that similar strata preserved within the GKP‐01 drill core through the Ghaap Group show pervasive remagnetisation by 2.5‐ to 1.8‐Ga nanoscale pyrrhotite. Furthermore, high‐resolution palaeomagnetic and geochemical evidence from GKF‐01 Neoarchaean nodular pyrite suggests that some of the Transvaal Supergroup pyrites have been post‐depositionally altered, ~0.5 Ga after their deposition (Fischer et al., 2014). Thus, even undeformed Ghaap Group strata can show a complex history of iron sulphide mineral precipitation.

**Figure 9:**
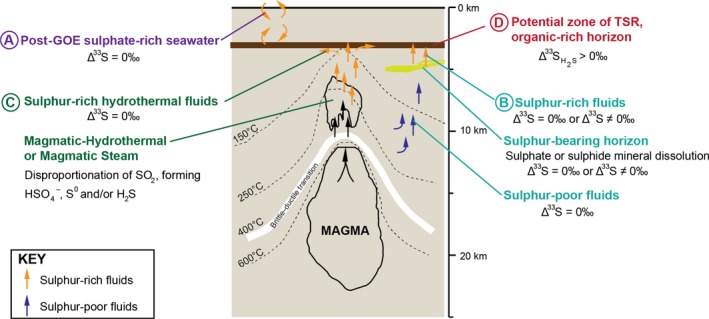
Hypothesised formation of post‐lithification pyrite with an anomalous Δ^33^S signal. Sulphur can be sourced via three pathways: (A) seawater, (B) dissolution of sulphate‐ or sulphide‐bearing minerals or (C) the disproportionation of SO_2_ in magmatic‐hydrothermal or magmatic steam environments

The geological evidence therefore suggests sulphur could have circulated in fluids as soluble sulphur species and participated in hydrothermal reactions in the Griqualand West Basin. There are multiple sources of subsurface S‐rich fluids (Figure 9): In modern settings, sulphate can be acquired from (buried) seawater (Ohmoto, 1972). If the hydrothermal alterations occurred after the GOE, seawater was a potential source (A, Figure 9). Furthermore, pore waters expelled from sedimentary rocks during compaction, fluids formed by magmatic/metamorphic processes or meteoric‐ or seawater‐derived fluids can cause the dissolution of S‐bearing minerals. Dissolution of sulphate, such as CAS or sulphate‐bearing minerals (e.g., gypsum, anhydrite or barite), occurs readily, while pyrite dissolution can also occur in the presence of an oxidant, for example, O_2_ or Fe^3+^ (Descostes, Vitorge, & Beaucaire, 2004; B, Figure 9). Moreover, both sulphate and sulphide can be produced from the disproportionation of magmatic SO_2_. For example, during the condensation of a magmatic plume, H_2_SO_4_ and H_2_S form from the disproportionation of magmatic SO_2_ at temperatures 200–400°C and pH < 3. Sulphate can be leached by fluids, causing the production of an acidic, sulphate‐bearing hydrothermal fluid that can infiltrate surrounding wall rock (C, Figure 9; Rye, 2005). The intrusion of the Bushveld Complex at ~2.06 Ga could have produced magmatic sulphur species (Hartzer, 1995). Finally, these subsurface S‐rich fluids could have infiltrated surrounding rocks via fracture flow or porous flow, and precipitated pyrite.

We suggest two scenarios whereby the circulation of S‐rich fluids from the above sources could have contributed to the formation of type 2 pyrite and its associated S‐MIF signal: one via thermochemical sulphate reduction of S‐bearing fluids from a more recent sulphur source, and one via migration of S‐rich fluids from stratigraphically older sediments carrying an inherited Archaean S‐MIF signal.

If the subsurface sulphur‐rich fluid were sourced from modern or post‐GOE seawater, the dissolution of post‐GOE sulphur minerals or magmatic sulphate, it would not carry a primary MIF signal. Therefore, to precipitate type 2 pyrite with a Δ^33^S fingerprint of +5.5‰ from one of these sources, a post‐depositional process would be necessary to cause S‐MIF. Experimental investigations have shown that thermochemical sulphate reduction (TSR) can produce a < 13‰ enrichment in Δ^33^S relative to the sulphate source (Oduro et al., 2011). An anomalous enrichment of ^33^S relative to ^34^S during TSR occurs due to magnetic isotope effects (MIE). In the sulphur isotope system, the ^33^S‐isotope contains magnetic nuclei; during TSR, ^33^S undergoes the spin‐selective reaction faster than ^34^S and ^32^S. This results in an anomalous enrichment of ^33^S relative to ^34^S in the polysulphide product, and therefore, the TSR product carries a positive Δ^33^S signal (Oduro et al., 2011).

Thermochemical sulphate reduction occurs at 100–300°C (Johnston, 2011); these temperatures are equivalent to sub‐greenschist to greenschist facies, which correspond to the maximum metamorphic grade that the lower Transvaal Supergroup, including BH1‐SACHA, experienced during intrusion of the Bushveld Complex in the Palaeoproterozoic (Sumner & Beukes, 2006). Therefore, the temperature regime during contact metamorphism of BH1‐SACHA and the surrounding rocks was sufficiently high to support TSR. The petrographic evidence and isotope fingerprint of type 2 pyrite, coupled with the history of hydrothermal alteration described above, could be consistent with MIE during TSR causing an anomalous enrichment in ^33^S relative to ^34^S. However, there is currently no evidence of anomalous Δ^33^S signals associated with TSR in bulk rock analyses from the geological record. Furthermore, TSR causes a MIF effect in ^33^S, but not ^36^S (Oduro et al., 2011); therefore, this hypothesis cannot be conclusively demonstrated without the inclusion of Δ^36^S data.

More likely, the MIF signal measured in type 2 pyrite could have formed from the dissolution and reprecipitation of Archaean S‐bearing minerals originally deposited with a positive Δ^33^S signature (Figure 9). Notably, bulk pyrite shows a Δ^33^S composition within error of the type 2 pyrite S‐isotope fingerprint, 20–30 m stratigraphically lower than sample‐3184 (Figure 2). Therefore, dissolution of these pyrite phases and migration of Δ^33^S = +5.5‰ sulphur‐rich fluids upwards are a likely source of the type 2 pyrite MIF signal. Therefore, grain‐scale Archaean MIF signatures may reflect secondary pyrite formation mechanisms as well as atmospheric processes at the time of deposition. These multiple pyrite phases can reflect a protracted history of pyrite precipitation, which can be recognised using high‐resolution, in situ S‐isotope geochemistry.

## 5 Conclusions

*Sulphate reduction in Neoarchaean microbial mats*: Our textural and sulphur isotope data echo other studies that suggest MSR influenced S‐cycling in the Neoarchaean (e.g., Habicht et al., 2002; Ueno et al., 2008; Zerkle, Claire, Domagal‐Goldman, Farquhar, & Poulton, 2012). As typical microbialite textures are generally associated with photoautotrophs (Noffke, 2009), this study proposes that microbial sulphate reducers may have commonly occurred in the same community as photosynthesisers, forming a microbial mat ecosystem similar to those of the modern.

*Neoarchaean seawater sulphate concentrations and S‐isotope composition*: SIMS S‐isotope data and closed‐system modelling suggest the local sulphur reservoir had a Δ^33^S = −0.21 ± 0.65‰ (±1σ, *n* = 177) and δ^34^S = 4‰–12‰ during formation of type 1 pyrites in sample‐3184 (Figure 8). These values are consistent with previous estimates of the δ^34^S‐isotope composition of Archaean sulphate (e.g., Domagal‐Goldman et al., 2008; Guo et al., 2009; Paris et al., 2014). However, the S‐isotope composition of seawater sulphate may have been spatially and temporally variable, particularly at the low sulphate levels estimated for the Neoarchaean (Zhelezinskaia et al., 2014). Closed‐system modelling of sample‐3184 indicates small MSR fractionation factors (α_MSR_ = 6‰–12‰), consistent with high reduction rates and low sulphate concentrations. The small MSR fractionation factors and scatter in Δ^33^S values in the literature imply sulphate had a short residence time in Archaean oceans due to a small reservoir size and comparatively large fluxes into and out of the reservoir. Additional studies assessing the S‐isotope composition of CAS will help constrain the evolution of seawater sulphate δ^34^S and Δ^33^S during the Archaean.

*The utility of combining petrography and SIMS*: This study demonstrates that grain‐scale MIF signals could record secondary processes in addition to contemporaneous atmospheric processes at the time of deposition. MIF signals can be generated by MIE during later TSR or, more probably, leached from surrounding Archaean sedimentary rocks and reprecipitated following lithification. Furthermore, it demonstrates that Archaean rocks can preserve a complex, protracted history of pyrite formation. Pyrite genesis can occur over several generations; precipitation can range from pre‐soft sediment deformation to post‐lithification. This study highlights the effectiveness of pairing petrography with high‐resolution SIMS sulphur isotope studies, to unravel the diagenetic processes that have contributed to multigenerational pyrite genesis.

## References

[gbi12227-bib-0001] Altermann, W. , & Siegfried, H. P. (1997). Sedimentology and facies development of an Archaean shelf: Carbonate platform transition in the Kaapvaal Craton, as deduced from a deep borehole at Kathu, South Africa. Journal of African Earth Sciences, 24, IN1–IN4.

[gbi12227-bib-0002] Altermann, W. , & Wotherspoon, J. M. (1995). The carbonates of the Transvaal and Griqualand West Sequences of the Kaapvaal craton, with special reference to the Lime Acres limestone deposit. Mineralium Deposita, 30, 124–134.

[gbi12227-bib-0003] Baroni, M. , Thiemens, M. H. , Delmas, R. J. , & Savarino, J. (2007). Mass‐independent sulfur isotopic compositions in stratospheric volcanic eruptions. Science, 315, 84–87. 17204647 10.1126/science.1131754

[gbi12227-bib-0004] Beukes, N. J. (1987). Facies relations, depositional environments and diagenesis in a major early Proterozoic stromatolitic carbonate platform to basinal sequence, Campbellrand Subgroup, Transvaal Supergroup, Southern Africa. Sedimentary Geology, 54, 1–46.

[gbi12227-bib-0005] Bradley, A. S. , Leavitt, W. D. , Schmidt, M. , Knoll, A. H. , Girguis, P. R. , & Johnston, D. T. (2016). Patterns of sulfur isotope fractionation during Microbial Sulfate Reduction. Geobiology, 14, 91–101. 26189479 10.1111/gbi.12149

[gbi12227-bib-0006] Button, A. (1973). Algal stromatolites of the early Proterozoic Wolkberg Group, Transvaal sequence. Journal of Sedimentary Research, 43, 160–167.

[gbi12227-bib-0007] Claire, M. W. , Kasting, J. F. , Domagal‐Goldman, S. D. , Stüeken, E. E. , Buick, R. , & Meadows, V. S. (2014). Modeling the signature of sulfur mass‐independent fractionation produced in the Archean atmosphere. Geochimica et Cosmochimica Acta, 141, 365–380.

[gbi12227-bib-0008] Crowe, S. A. , Paris, G. , Katsev, S. , Jones, C. , Kim, S. T. , Zerkle, A. L. , … Farquhar, J. (2014). Sulfate was a trace constituent of Archean seawater. Science, 346, 735–739. 25378621 10.1126/science.1258966

[gbi12227-bib-0009] De Kock, M. O. , Evans, D. A. D. , Kirschvink, J. L. , Beukes, N. J. , Rose, E. , & Hilburn, I. (2009). Paleomagnetism of a Neoarchean‐Paleoproterozoic carbonate ramp and carbonate platform succession (Transvaal Supergroup) from surface outcrop and drill core, Griqualand West region, South Africa. Precambrian Research, 169, 80–99.

[gbi12227-bib-0010] Descostes, M. , Vitorge, P. , & Beaucaire, C. (2004). Pyrite dissolution in acidic media. Geochimica et Cosmochimica Acta, 68, 4559–4569.

[gbi12227-bib-0011] Domagal‐Goldman, S. D. , Kasting, J. F. , Johnston, D. T. , & Farquhar, J. (2008). Organic haze, glaciations and multiple sulfur isotopes in the Mid‐Archean Era. Earth and Planetary Science Letters, 269, 29–40.

[gbi12227-bib-0012] Farquhar, J. , Bao, H. , & Thiemens, M. (2000). Atmospheric influence of Earth's earliest sulfur cycle. Science, 289, 756–758. 10926533 10.1126/science.289.5480.756

[gbi12227-bib-0013] Farquhar, J. , Cliff, J. , Zerkle, A. L. , Kamyshny, A. , Poulton, S. W. , Claire, M. , … Harms, B. (2013). Pathways for Neoarchean pyrite formation constrained by mass‐independent sulfur isotopes. Proceedings of the National Academy of Sciences, 110, 17638–17643. 10.1073/pnas.1218851110 PMC3816403 23407162

[gbi12227-bib-0014] Fike, D. A. , Bradley, A. S. , & Rose, C. V. (2015). Rethinking the ancient sulfur cycle. Annual Review of Earth and Planetary Sciences, 43, 593–622.

[gbi12227-bib-0015] Fike, D. A. , Finke, N. , Zha, J. , Blake, G. , Hoehler, T. M. , & Orphan, V. J. (2009). High resolution SIMS‐based sulfide δ^34^S: A new tool for characterizing microbial activity in a variety of depositional environments. Geochimica et Cosmochimica Acta, 73, 6187–6204.

[gbi12227-bib-0016] Fike, D. A. , Gammon, C. L. , Ziebis, W. , & Orphan, V. J. (2008). Micron‐scale mapping of sulfur cycling across the oxycline of a cyanobacterial mat: A paired nanoSIMS and CARD‐FISH approach. ISME Journal, 2, 749–759. 18528418 10.1038/ismej.2008.39

[gbi12227-bib-0017] Fischer, W. W. , Fike, D. A. , Johnson, J. E. , Raub, T. D. , Guan, Y. , Kirschvink, J. L. , & Eiler, J. M. (2014). SQUID–SIMS is a useful approach to uncover primary signals in the Archean sulfur cycle. Proceedings of the National Academy of Sciences, 111, 5468–5473. 10.1073/pnas.1322577111 PMC3992679 24706767

[gbi12227-bib-0019] Grobbelaar, W. S. , Burger, M. A. , Pretorius, A. I. , Marais, W. , & Van Niekerk, I. J. M. (1995). Stratigraphic and structural setting of the Griqualand West and the Olifantshoek Sequences at Black Rock, Beeshoek and Rooinekke Mines, Griqualand West, South Africa. Mineralium Deposita, 30, 152–161.

[gbi12227-bib-0020] Guo, Q. , Strauss, H. , Kaufman, A. J. , Schröder, S. , Gutzmer, J. , Wing, B. , … Farquhar, J. (2009). Reconstructing Earth's surface oxidation across the Archean‐Proterozoic transition. Geology, 37, 399–402.

[gbi12227-bib-0021] Habicht, K. S. , Gade, M. , Thamdrup, B. , Berg, P. , & Canfield, D. E. (2002). Calibration of sulfate levels in the Archean ocean. Science, 298, 2372–2374. 12493910 10.1126/science.1078265

[gbi12227-bib-0022] Hartzer, F. J. (1995). Transvaal Supergroup inliers: Geology, tectonic development and relationship with the Bushveld Complex, South Africa. Journal of African Earth Sciences, 21, 521–547.

[gbi12227-bib-0023] Huizenga, J. M. , Gutzmer, J. , Greyling, L. N. , & Schaefer, M. (2006). Carbonic fluid inclusions in Paleoproterozoic carbonate‐hosted Zn‐Pb deposits in Griqualand West, South Africa. South African Journal of Geology, 109, 55–62.

[gbi12227-bib-0024] Izon, G. , Zerkle, A. L. , Zhelezinskaia, I. , Farquhar, J. , Newton, R. J. , Poulton, S. W. , … Claire, M. W. (2015). Multiple oscillations in Neoarchaean atmospheric chemistry. Earth and Planetary Science Letters, 431, 264–273.

[gbi12227-bib-0025] Johnston, D. T. (2011). Multiple sulfur isotopes and the evolution of Earth's surface sulfur cycle. Earth‐Science Reviews, 106, 161–183.

[gbi12227-bib-0026] Johnston, D. T. , Farquhar, J. , Wing, B. A. , Kaufman, A. J. , Canfield, D. E. , & Habicht, K. S. (2005). Multiple sulfur isotope fractionations in biological systems: A case study with sulfate reducers and sulfur disproportionators. American Journal of Science, 305, 645–660.

[gbi12227-bib-0027] Kamber, B. S. , & Whitehouse, M. J. (2007). Micro‐scale sulphur isotope evidence for sulphur cycling in the late Archean shallow ocean. Geobiology, 5, 5–17. 10.1111/j.1472-4669.2006.00091.x 36298872

[gbi12227-bib-0028] Knoll, A. H. , & Beukes, N. J. (2009). Introduction: Initial investigations of a Neoarchean shelf margin‐basin transition (Transvaal Supergroup, South Africa). Precambrian Research, 169, 1–14.

[gbi12227-bib-0029] Laspidou, C. S. , & Rittmann, B. E. (2002). A unified theory for extracellular polymeric substances, soluble microbial products, and active and inert biomass. Water Research, 36, 2711–2720. 12146858 10.1016/s0043-1354(01)00413-4

[gbi12227-bib-0030] Martini, J. E. J. , Eriksson, P. G. , & Snyman, C. P. (1995). The early proterozoic Mississippi Valley‐type Pb‐Zn‐F deposits of the Campbellrand and Malmani subgroups, South Africa. Mineralium Deposita, 30, 135–145.

[gbi12227-bib-0031] Miyano, T. , & Beukes, N. J. (1984). Phase relations of stilpnomelane, ferri‐annite, and riebeckite in very low‐grade metamorphosed iron‐formations. South African Journal of Geology, 87, 111–124.

[gbi12227-bib-0032] Nielsen, P. H. , Jahn, A. , & Palmgren, R. (1997). Conceptual model for production and composition of exopolymers in biofilms. Water Science and Technology, 36, 11–19.

[gbi12227-bib-0033] Noffke, N. (2009). The criteria for the biogeneicity of microbially induced sedimentary structures (MISS) in Archean and younger, sandy deposits. Earth‐Science Reviews, 96, 173–180.

[gbi12227-bib-0034] Oduro, H. , Harms, B. , Sintim, H. O. , Kaufman, A. J. , Cody, G. , & Farquhar, J. (2011). Evidence of magnetic isotope effects during thermochemical sulfate reduction. Proceedings of the National Academy of Sciences, 108, 17635–17638. 10.1073/pnas.1108112108 PMC3203815 21997216

[gbi12227-bib-0035] Ohmoto, H. (1972). Systematics of sulfur and carbon isotopes in hydrothermal ore deposits. Economic Geology, 67, 551–578.

[gbi12227-bib-0036] Ono, S. , Beukes, N. J. , & Rumble, D. (2009). Origin of two distinct multiple‐sulfur isotope compositions of pyrite in the 2.5 Ga Klein Naute Formation, Griqualand West Basin, South Africa. Precambrian Research, 169, 48–57.

[gbi12227-bib-0037] Ono, S. , Eigenbrode, J. L. , Pavlov, A. A. , Kharecha, P. , Rumble, D. , Kasting, J. F. , & Freeman, K. H. (2003). New insights into Archean sulfur cycle from mass‐independent sulfur isotope records from the Hamersley Basin, Australia. Earth and Planetary Science Letters, 213, 15–30.

[gbi12227-bib-0038] Paris, G. , Adkins, J. F. , Sessions, A. L. , Webb, S. M. , & Fischer, W. W. (2014). Neoarchean carbonate–associated sulfate records positive Δ33S anomalies. Science, 346, 739–741. 25378622 10.1126/science.1258211

[gbi12227-bib-0039] Pavlov, A. A. , & Kasting, J. F. (2002). Mass‐independent fractionation of sulfur isotopes in Archean sediments: Strong evidence for an anoxic Archean atmosphere. Astrobiology, 2, 27–41. 12449853 10.1089/153110702753621321

[gbi12227-bib-0040] Paytan, A. , Mearon, S. , Cobb, K. , & Kastner, M. (2002). Origin of marine barite deposits: Sr and S isotope characterization. Geology, 30, 747–750.

[gbi12227-bib-0041] Peres, P. , de Chambost, E. , & Schuhmacher, M. (2008). CAMECA IMS 7f‐Geo: Specialized SIMS tool for geosciences. Applied Surface Science, 255, 1472–1475.

[gbi12227-bib-0042] Roerdink, D. L. , Mason, P. R. , Farquhar, J. , & Reimer, T. (2012). Multiple sulfur isotopes in Paleoarchean barites identify an important role for microbial sulfate reduction in the early marine environment. Earth and Planetary Science Letters, 331, 177–186.

[gbi12227-bib-0043] Rye, R. O. (2005). A review of the stable‐isotope geochemistry of sulfate minerals in selected igneous environments and related hydrothermal systems. Chemical Geology, 215, 5–36.

[gbi12227-bib-0044] Schieber, J. (1999). Microbial mats in terrigenous clastics; the challenge of identification in the rock record. Palaios, 14, 3–12.

[gbi12227-bib-0045] Schieber, J. (2007). Microbial mats on muddy substrates—examples of possible sedimentary features and underlying processes. Atlas of Microbial Mat Features Preserved within the Siliciclastic Rock Record Atlases in Geoscience, 2, 117–133.

[gbi12227-bib-0046] Schröder, S. , Lacassie, J. P. , & Beukes, N. J. (2006). Stratigraphic and geochemical framework of the Agouron drill cores, Transvaal Supergroup (Neoarchean–Paleoproterozoic, South Africa). South African Journal of Geology, 109, 23–54.

[gbi12227-bib-0047] Shen, Y. , & Buick, R. (2004). The antiquity of microbial sulfate reduction. Earth‐Science Reviews, 64, 243–272.

[gbi12227-bib-0048] Shen, Y. , Buick, R. , & Canfield, D. E. (2001). Isotopic evidence for microbial sulphate reduction in the early Archaean era. Nature, 410, 77–81. 11242044 10.1038/35065071

[gbi12227-bib-0049] Sumner, D. Y. , & Beukes, N. J. (2006). Sequence stratigraphic development of the Neoarchean Transvaal carbonate platform, Kaapvaal Craton, South Africa. South African Journal of Geology, 109, 11–22.

[gbi12227-bib-0050] Ueno, Y. , Ono, S. , Rumble, D. , & Maruyama, S. (2008). Quadruple sulfur isotope analysis of ca. 3.5 Ga Dresser Formation: New evidence for microbial sulfate reduction in the early Archean. Geochimica et Cosmochimica Acta, 72, 5675–5691.

[gbi12227-bib-0051] Van Kranendonk, M. J. (2006). Volcanic degassing, hydrothermal circulation and the flourishing of early life on Earth: A review of the evidence from c. 3490–3240 Ma rocks of the Pilbara Supergroup, Pilbara Craton, Western Australia. Earth‐Science Reviews, 74, 197–240.

[gbi12227-bib-0052] Wagner, M. , Roger, A. J. , Flax, J. L. , Brusseau, G. A. , & Stahl, D. A. (1998). Phylogeny of dissimilatory sulfite reductases supports an early origin of sulfate respiration. Journal of Bacteriology, 180, 2975–2982. 9603890 10.1128/jb.180.11.2975-2982.1998 PMC107267

[gbi12227-bib-0053] Wilbanks, E. G. , Jaekel, U. , Salman, V. , Humphrey, P. T. , Eisen, J. A. , Facciotti, M. T. , … Orphan, V. J. (2014). Microscale sulfur cycling in the phototrophic pink berry consortia of the Sippewissett Salt Marsh. Environmental Microbiology, 16, 3398–3415. 24428801 10.1111/1462-2920.12388 PMC4262008

[gbi12227-bib-0054] Zerkle, A. L. , Claire, M. W. , Domagal‐Goldman, S. D. , Farquhar, J. , & Poulton, S. W. (2012). A bistable organic‐rich atmosphere on the Neoarchaean Earth. Nature Geoscience, 5, 359–363.

[gbi12227-bib-0055] Zhelezinskaia, I. , Kaufman, A. J. , Farquhar, J. , & Cliff, J. (2014). Large sulfur isotope fractionations associated with Neoarchean microbial sulfate reduction. Science, 346, 742–744. 25378623 10.1126/science.1256211

