# Supplementary material for: Sulphur cycling in a Neoarchaean microbial mat
Source: Geobiology. 2017 Jan 27;15(3):353–65. doi: 10.1111/gbi.12227 (PMC5412852; doi:10.1111/gbi.12227)
Supplement: Supplementary file 1 [file GBI-15-353-s001.pdf]

SUPPLEMENTARY MATERIALS

Determining the corrected isotopic composition of the in-house standard

The in-house standard was analysed 12 times and bracketed by a total of 20 runs of UAriz 127-6 Balmat standard on the CAMECA IMS 7f-GEO Secondary Ion Mass Spectrometer (SIMS) housed in the Department of Earth and Planetary Sciences at Washington University (St Louis, MO, USA). The Mesoproterozoic Balmat massive sulphide deposit was sampled from the Adirondack Mountains, New York, USA and has experienced upper Amphibolite facies metamorphism (Crowe and Vaughan, 1996; Ushikubo et al., 2014). The $\delta^{34}S_{V\text{-}CDT}$ value of the Balmat standard was independently determined by EA-IRMS to be 15.1‰ (consistent with other reports of Balmat $\delta^{34}S$ values). The Balmat standard's $\delta^{33}S_{V\text{-}CDT}$ composition of 7.75‰ was deduced using the $\delta^{33}S/\delta^{34}S$ = 0.513 ratio (Ushikubo, 2014). Assuming a V-CDT composition of $^{32}S/^{33}S$ = 126.948 and $^{32}S/^{34}S$ = 22.643 (Ding et al., 2001), the calculated Balmat standard ^{33}R and ^{34}R values are 0.007938 and 0.044831, respectively.

Correction factors for the Balmat standard are ratios that relate the accepted ^{33}R and ^{34}R values ($^{33}R_{(Balmat,\ known)}$ and $^{34}R_{(Balmat,\ known)}$) with the mean of 20 SIMS measured values ($^{33}R_{(Balmat,\ meas)}$ and $^{34}R_{(Balmat,\ meas)}$; Equations S1)–S2). Table S1 shows a summary of the measured sulphur compositions of UAriz 127-6. The correction factors ($^{33}C_{Balmat}$ and $^{34}C_{Balmat}$) are 0.9980 and 0.9986 for ^{33}R and ^{34}R, respectively.

Table S1. Summary of 20 S-isotope measurements of UAriz 127-6 Balmat standard on the SIMS

	^{33}**R**	^{34}**R**
**Mean**	0.0079544	0.044895
**Standard deviation**	0.0000039	0.000039
**Relative standard deviation (‰)**	0.49	0.87
**Relative standard error (‰)**	0.11	0.19

$$\text{Balmat correction factor } (^{33}C_{Balmat}) = \frac{^{33}R_{(Balmat,\ known)}}{^{33}R_{(Balmat,\ meas)}} \qquad \text{(S1)}$$

$$\text{Balmat correction factor } (^{34}C_{Balmat}) = \frac{^{34}R_{(Balmat,\ known)}}{^{34}R_{(Balmat,\ meas)}} \quad \text{(S2)}$$

The accepted value of the in-house standard's ^{33}R and ^{34}R ratios ($^{33}R_{std}$ and $^{34}R_{std}$) were calculated by applying the Balmat correction factors ($^{33}C_{Balmat}$ and $^{34}C_{Balmat}$) to the mean of the in-house standard measured ratios (n = 12; $^{33}R_{(std,\ meas)}$ and $^{34}R_{(std,\ meas)}$; Table S2; Equations S3–S4). The accepted composition of the in-house standard ($^{33}R_{std}$ and $^{34}R_{std}$) are 0.007878 and 0.04417 for ^{33}R and ^{34}R, respectively.

Table S2. Summary of 12 S-isotope measurements of the in-house standard via SIMS

	^{33}R	^{34}R
**Mean**	0.0078943	0.044233
**Standard deviation**	0.0000048	0.000040
**Relative standard deviation (‰)**	0.61	0.91
**Relative standard error (‰)**	0.18	0.26

$$^{33}R_{std} = {}^{33}C_{Balmat} \times {}^{33}R_{(std,\ meas)} \quad \text{(S3)}$$

$$^{34}R_{std} = {}^{34}C_{Balmat} \times {}^{34}R_{(std,\ meas)} \quad \text{(S4)}$$

Subsequently, the accepted $\delta^{33}S_{V\text{-}CDT}$ and $\delta^{34}S_{V\text{-}CDT}$ were obtained using Equations 1–2 and the V-CDT composition determined by Ding et al. (2001; Table S3), and supported by bulk IRMS analyses.

Table S3. Summary of the in-house standard's corrected S-isotope composition, relative to V-CDT

$\delta^{34}S_{V\text{-}CDT}$	$\delta^{33}S_{V\text{-}CDT}$	^{34}R	^{33}R
0.13	0.13	0.04417	0.007878

The internal precision of the Balmat and in-house standard bracketing runs was ~0.2‰ for ^{33}R and ^{34}R (1 standard error). External precision, assessed by multiple replicate analysis on the

Balmat and in-house standards were typically 0.38‰ and 0.32‰ for $\delta^{34}S$ and $\delta^{33}S$, respectively.

Normalising procedure

An average analysis session of ~50 unknowns was bracketed by ≥4 measurements of the in-house standard; a mean S-isotope composition of the in-house standard was calculated for the start and end of each session ($^{33}R_{(std, meas)}$ and $^{34}R_{(std, meas)}$). Correction factors for the mean of the in-house standard ($^{33}C_{std}$ and $^{34}C_{std}$) at the start and end of a session were obtained using Equation S5–S6, where the accepted R values of the standard ($^{33}R_{std}$ and $^{34}R_{std}$) are shown in Table S3.

$$^{33}C_{std} = \frac{^{33}R_{std}}{^{33}R_{(std,\ meas)}} \tag{S5}$$

$$^{34}C_{std} = \frac{^{34}R_{std}}{^{34}R_{(std,\ meas)}} \tag{S6}$$

Correction factors for each unknown sample ($^{33}C_{unknown}$ and $^{34}C_{unknown}$) were calculated using the correction factors of the in-house standard at the beginning and end of each analysis session ($^{33}C_{(std, init)}$ and $^{34}C_{(std, final)}$) and by assuming a linear regression in the case of an offset. This accounts for the linear drift in the instrument during a session (Equations S7–S8).

$$^{33}C_{unknown} = \frac{^{33}C_{(std,\ init.)} - {}^{33}C_{(std,\ final)}}{\text{no. of unknowns in session} + 1} \times \text{analysis no. within session} \tag{S7}$$

$$^{34}C_{unknown} = \frac{^{34}C_{(std,\ init.)} - {}^{34}C_{(std,\ final)}}{\text{no. of unknowns in session} + 1} \times \text{analysis no. within session} \tag{S8}$$

The corrected ^{33}R and ^{34}R ratios for the unknown samples ($^{33}R_{(unknown, corr)}$ and $^{34}R_{(unknown, corr)}$) were obtained using the following equations:

$$^{33}R_{(unknown,\ corr)} = {}^{33}R_{(unknown,\ meas)} \times {}^{33}C_{unknown} \qquad (S9)$$

$$^{34}R_{(unknown,\ corr)} = {}^{34}R_{(unknown,\ meas)} \times {}^{34}C_{unknown} \qquad (S10)$$

Finally, the corrected R values ($^{33}R_{(unknown,\ corr)}$ and $^{34}R_{(unknown,\ corr)}$) were used to determine the $\delta^{34}S_{V\text{-}CDT}$, $\delta^{33}S_{V\text{-}CDT}$ and therefore, $\Delta^{33}S$ of the unknown (Equations 1–3).

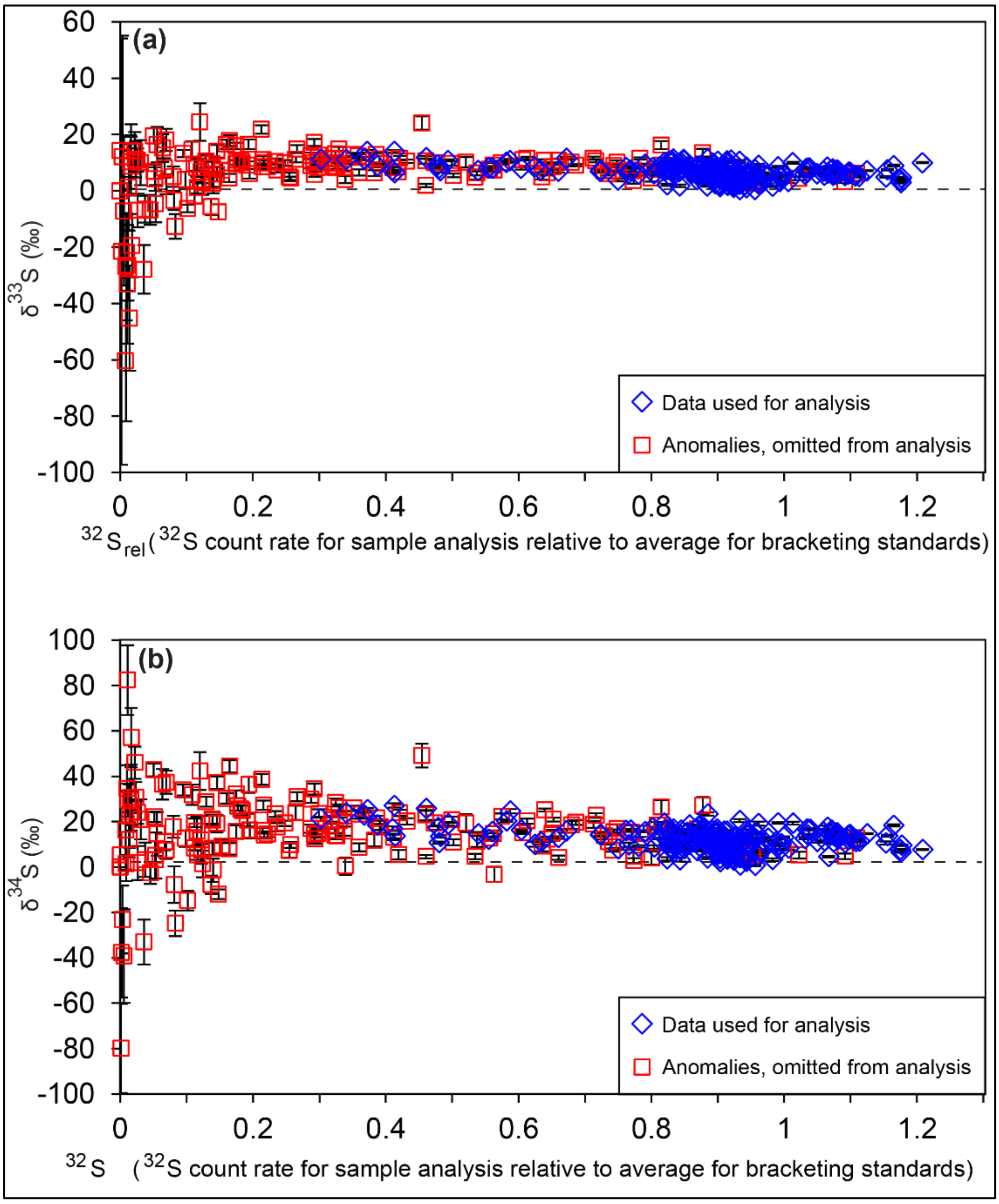

Fig. S1. A plot to show $^{32}S_{rel}$ against **(a)** $\delta^{33}S$ (‰), and **(b)** $\delta^{34}S$ (‰). $^{32}S_{rel}$ is the count rate for sample/unknown analysis relative to the average for the bracketing standards. Both delta values are relative to V-CDT. A low sulphide concentration for the sample would result in a lower $^{32}S_{rel}$ and large error bars. Error bars represent 1SE of each measurement (n = 15 cycles). This plot contains all data before omission of anomalies.

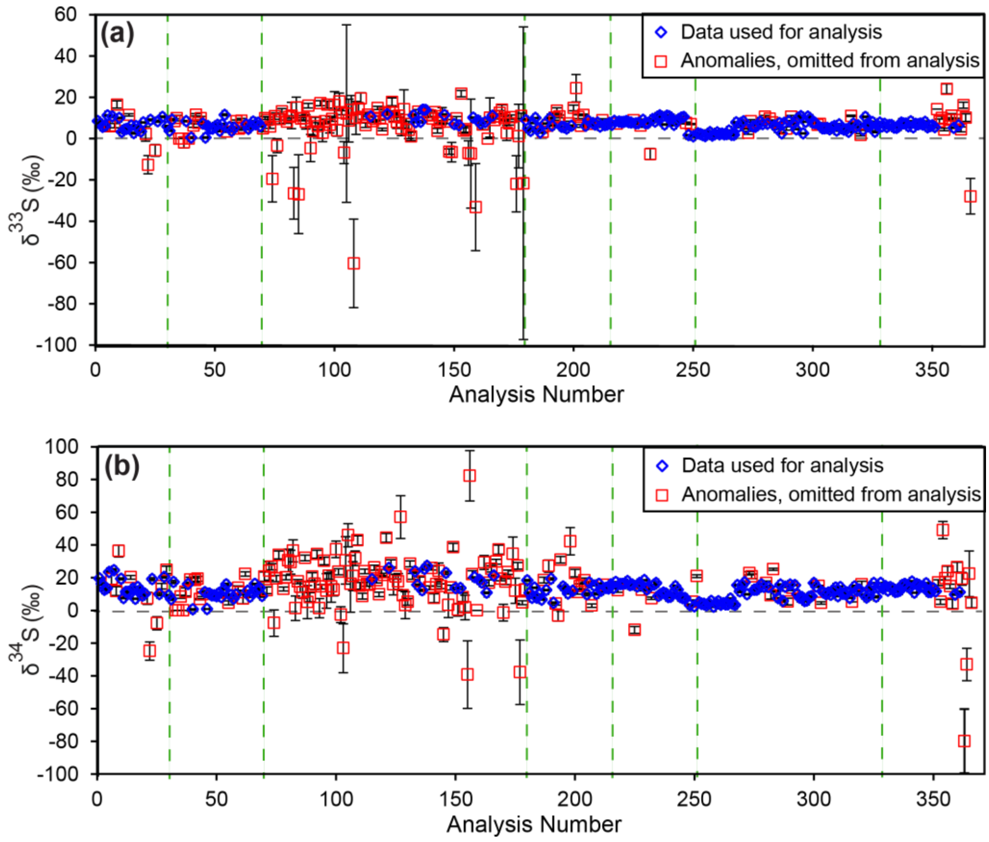

Fig. S2. A plot to show the analysis number of unknowns against **(a)** $\delta^{33}S$ (‰), and **(b)** $\delta^{34}S$ (‰). Both delta values are relative to V-CDT. Standards were analysed at the green dashed lines, separating sessions. Error bars represent 1SE of each measurement (n = 15 cycles). This plot contains all data before omission of anomalies. Note the third session (analysis numbers 70-177) has particularly poor precision. During this session, a transect parallel to stratigraphic height was attempted. However, due to a poor spot analysis selection, a consistently high matrix to sulphide ratio in the analysis spots caused large uncertainties. Most of these measurements are anomalous and therefore were not further considered.

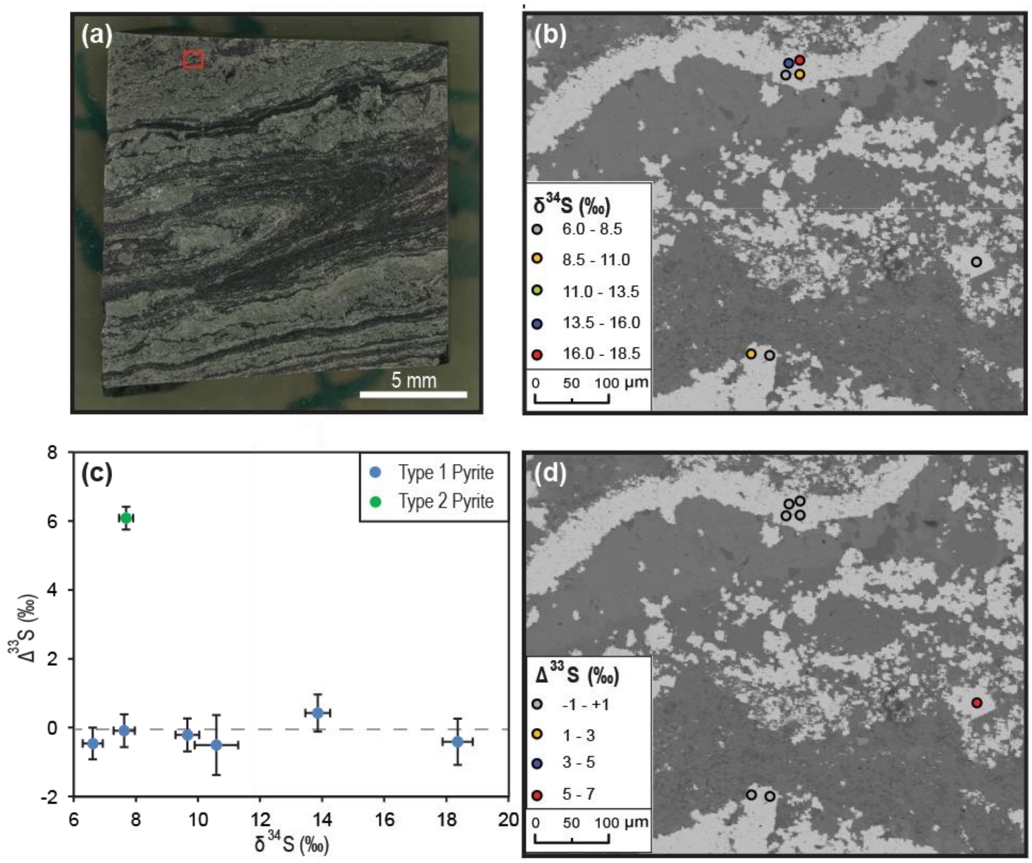

Fig. S3. Multiple SIMS S-isotope data of wavy crinkled lamination composed of type 1 pyrite. **(a)** Reflected light photomicrograph showing the location of BSE images (b) and (d) relative to the thick section. **(b)** SIMS $\delta^{34}S$ data overlain on a BSE image of the analysis region. **(c)** Plot to show SIMS $\delta^{34}S$ against $\Delta^{33}S$ data (‰). Blue circles = type 1 pyrite, green circles = type 2 pyrite. Error bars represent 1SE for each measurement (n = 15 cycles). **(d)** SIMS $\Delta^{33}S$ data overlain on a BSE image of the analysis region. As the characteristic type 1 pyrite S-isotope composition is observed in wavy crinkled lamination, a typical biogenic textural feature of microbialites, type 1 pyrite captures an early-diagenetic signal.

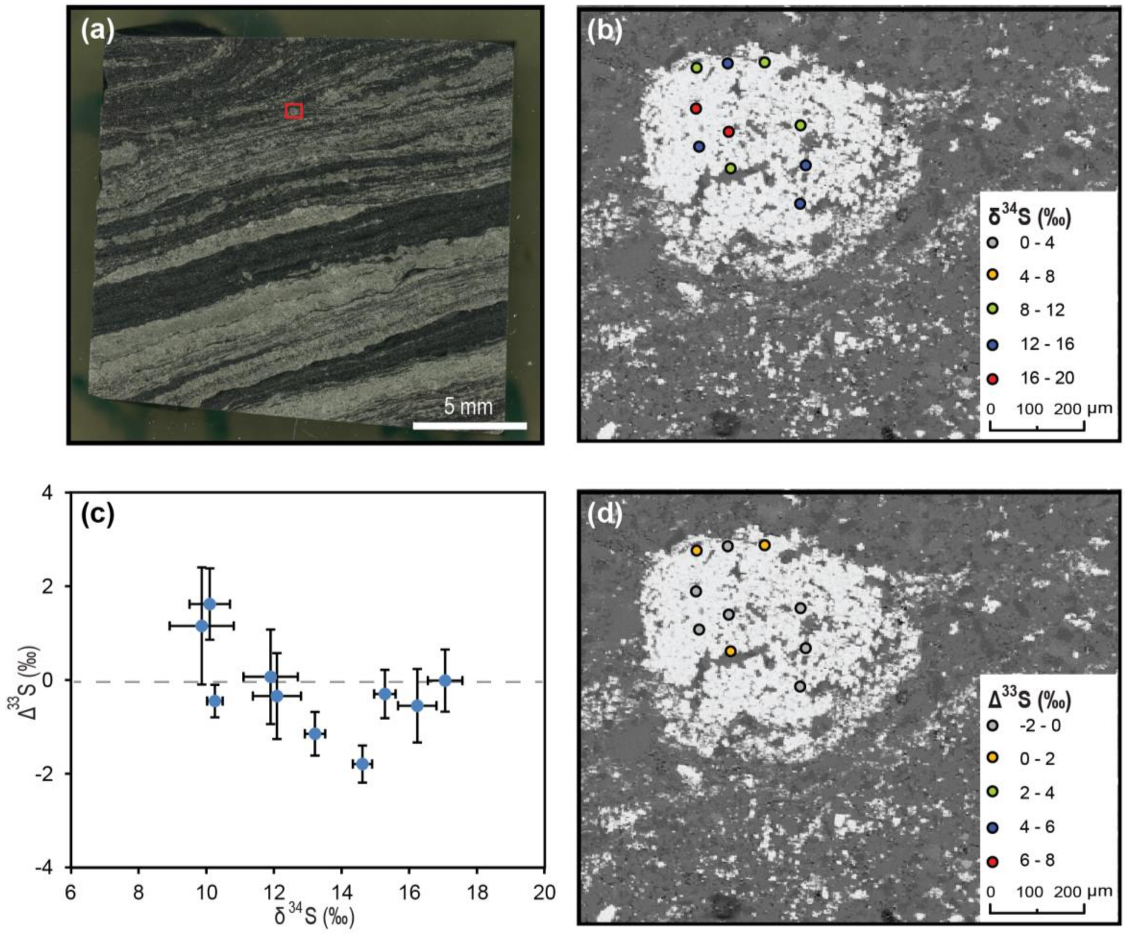

Fig. S4. Multiple S-isotope studies of a post-depositional, pre-lithification concretion composed of type 1 pyrite. **(a)** Reflected light photomicrograph showing the location of BSE images B and D relative to the thick section. **(b)** SIMS $\delta^{34}S$ data overlain on a BSE image of the pyrite concretion. **(c)** Plot to show SIMS $\delta^{34}S$ against $\Delta^{33}S$ data (‰). Error bars represent 1SE for each measurement (n = 15 cycles). **(d)** SIMS $\Delta^{33}S$ data overlain on a BSE image of the pyrite concretion. The concretion cross-cuts and causes the deformation of laminae; therefore, it formed after deposition but before lithification. This petrographic evidence suggests type 1 pyrite formed during early-diagenesis.

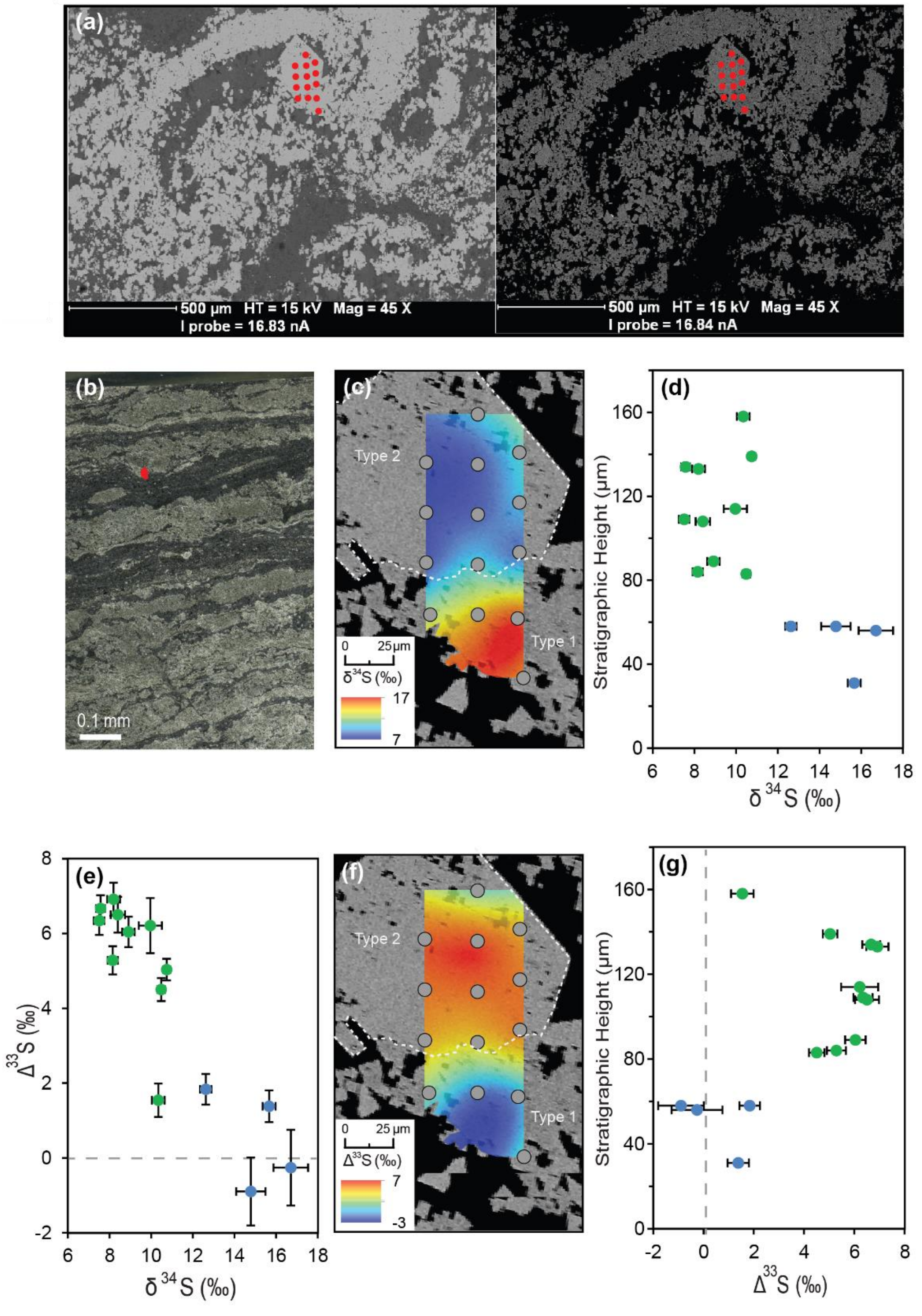

Fig. S5. An example of the contrasting textural and S-isotope signatures of type 1 and type 2 pyrite. **(a)** BSE images of the analytical grid location. There is a ~250 μm type 2 pyrite grains in the centre of type 1 pyrite aggregates. **(b)** Reflected light photomicrograph of the analysis area within a pyrite-rich lamina. **(c)** BSE image of the analysis area overlain by a SIMS $\delta^{34}S$ image constructed by spline interpolation of the analytical grid (n = 14). **(d)** SIMS $\delta^{34}S$ data (‰) against stratigraphic height (μm). The stratigraphic height corresponds to the BSE image in (c). **(e)** Plot to show SIMS $\delta^{34}S$ against $\Delta^{33}S$ data (‰). **(f)** BSE image of the analysis area overlain by a SIMS $\Delta^{33}S$ image constructed by spline interpolation of the analytical grid (n = 14). **(g)** SIMS $\Delta^{33}S$ data (‰) against stratigraphic height (μm). The stratigraphic height corresponds to the BSE image in (f). All error bars in (d), (e) and (g) plots represent 1SE for each measurement (n = 15 cycles). Blue circles = type 1 pyrite, green circles = type 2 pyrite.

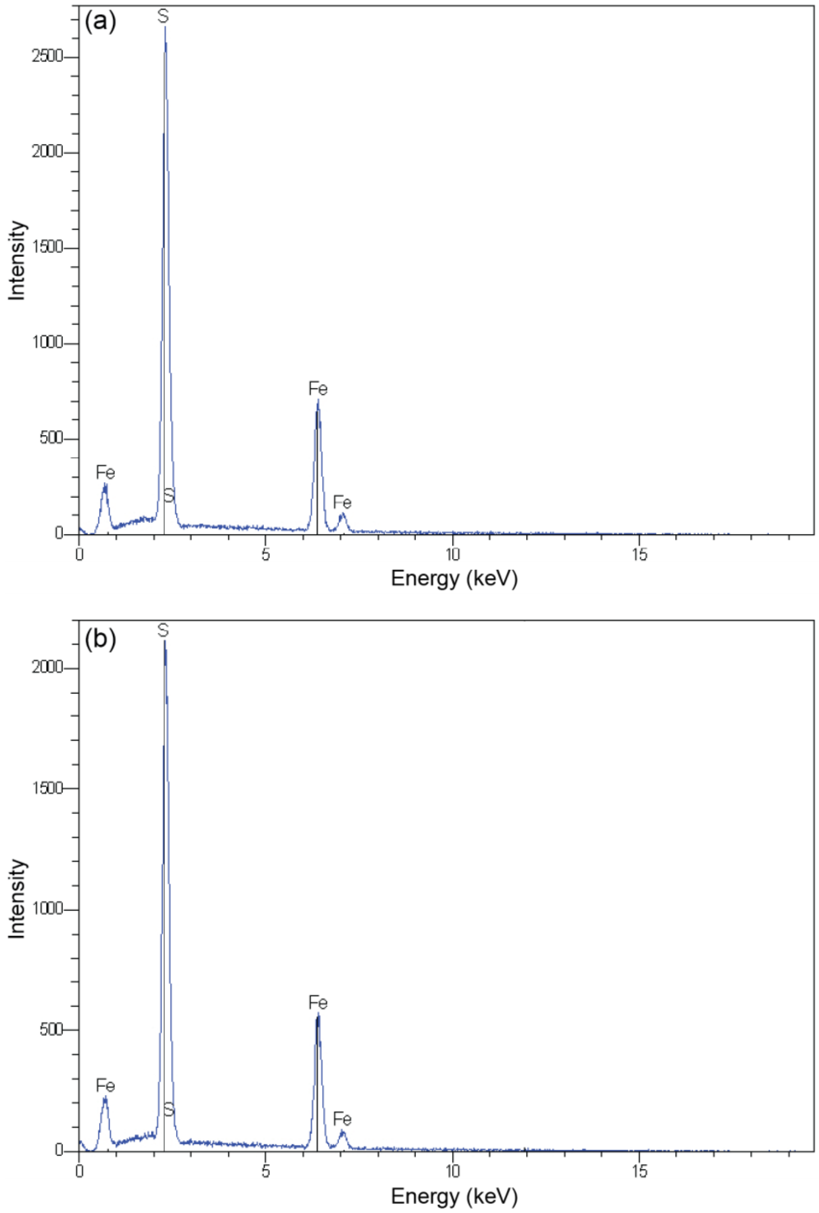

Fig. S6. EPMA plots showing elemental compositions of type 1 pyrite (a) and a mixture of type 1 and type 2 pyrite (b).

Table S4. Multiple sulphur isotope data, collected *in-situ* using secondary ion mass spectrometry (SIMS), from pyrite within a ~2.65 Ga microbialite, Lokammona Formation, Ghaap Group, South Africa

Sample ID	Measured ^{32}Sx10^{8} (cps)	$\delta^{33}S_{V\text{-}CDT}$ (‰)	1SE (‰)	$\delta^{34}S_{V\text{-}CDT}$ (‰)	1SE (‰)	$\Delta^{33}S_{V\text{-}CDT}$ (‰)	1SE (‰)	Pyrite Type*
NM_1c@1	1.94	8.53	0.31	19.4	0.39	-1.44	0.55	1
NM_1c@2	1.50	7.45	0.50	16.2	0.71	-0.85	0.96	1
NM_1c@3	1.91	5.70	0.35	12.7	0.69	-0.81	0.87	1
NM_1c@4	1.77	6.19	0.30	13.4	0.55	-0.68	0.69	1
NM_1c@5	1.73	11.30	0.28	23.4	0.71	-0.69	0.85	1
NM_1c@7	1.15	10.76	0.56	24.6	0.96	-1.83	1.25	1
NM_1c@10	1.60	10.05	0.31	19.3	0.56	0.15	0.71	1
NM_1d@1	1.94	3.29	0.31	7.6	0.45	-0.62	0.61	1
NM_1d@2	1.93	4.41	0.48	11.3	0.80	-1.40	1.04	1
NM_1d@3	1.84	4.73	0.45	11.2	0.92	-1.04	1.15	1
NM_1d@5	1.81	6.34	0.38	13.3	0.58	-0.49	0.76	1
NM_1d@6	1.89	2.64	0.41	7.1	0.33	-1.02	0.54	1
NM_1d@7	1.85	6.07	0.29	14.2	0.49	-1.21	0.63	1
NM_1d@8	1.87	4.78	0.37	12.5	0.54	-1.65	0.73	1
NM_1d@9	1.85	3.46	0.32	9.0	0.52	-1.14	0.68	1
NM_1e@1	1.66	7.85	0.27	10.1	0.58	2.67	0.71	1 and 2
NM_1e@4	1.88	9.08	0.46	19.1	0.89	-0.71	1.13	1
NM_1e@7	1.22	8.41	0.50	10.0	1.03	3.27	1.28	1 and 2
NM_1e@9	1.83	10.54	0.39	20.5	0.65	0.05	0.84	1
NM4pos_a_x-2717y-1102	1.28	5.93	0.53	9.2	0.52	1.23	0.79	1
NM4pos_a_x-2729y-1051	1.58	3.82	0.28	6.9	0.70	0.29	0.84	1
NM4pos_a_x-2722y-970	0.79	9.22	0.42	17.4	0.97	0.32	1.18	1
NM4pos_a_x-2689y-778a	1.55	8.48	0.31	16.0	0.58	0.26	0.74	1
NM4pos_a_x-2910y-538	1.57	0.48	0.44	0.7	0.33	0.12	0.55	1
NM4pos_a_x-2920y-874	1.56	3.40	0.34	6.9	0.35	-0.14	0.52	1
NM4pos_a_x-2959y-1159	1.04	7.25	0.61	11.1	1.06	1.53	1.37	1
NM4pos_b_x1762y3452	1.53	0.53	0.34	0.9	0.20	0.09	0.37	1
NM4pos_b_x1762y3515	1.52	6.06	0.16	10.3	0.20	0.75	0.28	1
NM4pos_b_x1762y3578	1.53	4.90	0.33	9.2	0.43	0.16	0.60	1
NM4pos_b_x1762y3641	1.53	4.92	0.42	9.2	0.77	0.17	0.98	1
NM4pos_b_x1762y3704	1.44	5.85	0.46	8.7	0.82	1.38	1.05	1
NM4pos_b_x1762y3767	1.53	6.67	0.28	10.6	0.36	1.21	0.50	1
NM4pos_b_x1762y3830	1.23	3.82	0.45	8.0	0.40	-0.30	0.63	1
NM4pos_b_x1762y3893	1.08	6.94	0.47	13.0	0.54	0.26	0.78	1
NM4pos_b_x1712y3830	1.10	11.55	0.46	15.9	0.77	3.40	1.00	1 and 2
NM4pos_b_x1702y3718	1.50	5.56	0.47	9.4	0.60	0.72	0.83	1
NM4pos_b_x1710y3646	1.51	3.83	0.32	7.2	0.25	0.12	0.41	1
NM4pos_b_x1710y3583	1.49	5.30	0.29	9.6	0.31	0.37	0.46	1
NM4pos_b_x1665y3678	1.42	6.91	0.32	12.0	0.36	0.76	0.52	1
NM4pos_b_x1665y3741	1.37	4.75	0.39	8.4	0.44	0.44	0.63	1
NM4pos_b_x1665y3804	1.47	6.11	0.40	11.1	0.37	0.42	0.57	1
NM4pos_b_x2499y4066	1.25	8.32	0.45	16.2	0.41	0.02	0.63	1

NM4pos_b_x2511y4139	1.44	6.90	0.37	12.7	0.60	0.40	0.79	1
NM4pos_b_x2515y4234	1.46	7.78	0.38	13.7	0.28	0.74	0.47	1
NM4pos_b_x2519y4316	1.32	6.09	0.42	8.8	0.78	1.55	0.99	1
nm_x2904y-81	1.08	10.83	0.37	19.0	0.83	1.07	1.02	1
nm_x2886y139	1.01	11.88	0.54	25.8	1.08	-1.33	1.35	1
nm_x2582y3261	0.74	11.23	0.69	23.9	0.78	-1.01	1.12	1
nm_x2585y3383	1.22	7.98	0.27	15.6	0.63	0.00	0.76	1
nm_x2585y3472	1.21	8.20	0.60	12.3	0.60	1.90	0.90	1
nm_x2585y3529	0.81	14.10	0.47	25.4	0.66	1.10	0.89	1
nm_x2579y3544	0.90	13.97	0.49	27.1	0.97	0.08	1.22	1
nm_x1903y4577	0.80	11.47	0.57	23.0	1.01	-0.28	1.29	1
nm_x1927y4721	1.05	7.08	0.54	13.5	0.55	0.14	0.82	1
nm_x1904y4809	0.90	6.45	0.65	13.7	0.98	-0.57	1.30	1
nm_x1909y4954	1.27	10.17	0.31	20.3	0.29	-0.22	0.45	1
nm_x1940y5050	0.85	8.92	0.54	18.6	0.41	-0.61	0.69	1
nm_x1940y5112	0.90	7.40	1.00	17.0	1.01	-1.30	1.51	1
nm_x1945y5210	1.05	8.02	0.37	10.8	0.50	2.49	0.68	1 and 2
nm_x1982y9372	0.66	11.16	0.75	21.2	0.96	0.28	1.33	1
NM4_serp@3	2.59	9.00	0.37	18.4	0.49	-0.41	0.67	1
NM4_serp@4	2.57	4.94	0.34	10.6	0.70	-0.51	0.87	1
NM4_serp@5	2.62	3.83	0.27	7.6	0.34	-0.09	0.48	1
NM4_serp@6	2.57	7.54	0.28	13.9	0.40	0.42	0.54	1
NM4_serpu@1	2.69	10.04	0.21	7.7	0.23	6.08	0.33	2
NM4_serpu@2	2.62	4.75	0.19	9.7	0.38	-0.21	0.48	1
NM4_serpu@3	2.62	2.94	0.27	6.6	0.32	-0.46	0.46	1
NM4_ne@2	2.41	7.26	0.27	12.4	0.39	0.88	0.52	1
NM4_ne@4	2.26	9.95	0.36	19.3	0.63	0.05	0.81	1
NM4_ne@5	2.38	6.35	0.30	4.6	0.40	3.99	0.55	1 and 2
NM2_BALL@1	2.13	5.71	0.22	14.6	0.29	-1.79	0.40	1
NM2_BALL@02	2.23	5.64	0.33	13.2	0.30	-1.15	0.47	1
NM2_BALL@03	2.22	4.82	0.24	10.3	0.23	-0.45	0.35	1
NM2_BALL@05	2.16	6.18	0.41	11.9	0.80	0.07	1.01	1
NM2_BALL@11	1.71	6.22	0.60	9.9	0.95	1.15	1.25	1
NM2_BALL@12	1.93	8.74	0.30	17.1	0.51	-0.01	0.66	1
NM2_BALL@14	2.13	7.54	0.40	15.3	0.32	-0.30	0.52	1
NM2_BALL@15	1.91	6.81	0.32	10.1	0.60	1.62	0.76	1
NM2_BALL@16	1.88	7.78	0.43	16.2	0.57	-0.55	0.79	1
NM2_BALL@17	1.95	5.86	0.41	12.1	0.71	-0.34	0.92	1
NM3_OZ@1	1.91	6.58	0.31	13.7	0.23	-0.46	0.39	1
NM3_OZ@02	1.88	6.77	0.27	13.4	0.33	-0.11	0.46	1
NM3_OZ@03	1.90	7.65	0.40	15.9	0.30	-0.53	0.50	1
NM3_OZ@04	1.91	6.75	0.33	13.6	0.28	-0.24	0.44	1
NM3_OZ@05	1.86	8.60	0.17	17.4	0.25	-0.33	0.34	1
NM3_OZ@07	1.89	8.31	0.28	16.1	0.27	0.05	0.41	1
NM3_OZ@08	1.28	8.01	0.45	16.2	0.43	-0.32	0.65	1
NM3_OZ@09	1.84	8.15	0.30	16.5	0.35	-0.32	0.50	1
NM3_OZ@10	1.85	8.45	0.31	17.8	0.24	-0.70	0.40	1
NM3_OZ@11	1.81	7.98	0.29	15.4	0.29	0.10	0.44	1

NM3_OZ@12	1.76	7.84	0.31	15.8	0.49	-0.26	0.65	1
NM3_OZ@14	1.53	7.36	0.28	14.6	0.64	-0.11	0.78	1
NM3_OZ@15	1.74	7.54	0.39	16.6	0.57	-1.00	0.76	1
NM3_OZ@17	1.82	9.14	0.28	18.5	0.29	-0.35	0.43	1
NM3_OZ@18	1.75	7.59	0.35	12.7	0.45	1.05	0.62	1 and 2
NM3_OZ@19	1.79	7.48	0.27	14.4	0.32	0.08	0.46	1
NM3_uhed@1	1.68	9.42	0.23	15.7	0.31	1.38	0.42	1
NM3_uhed@02	1.77	8.31	0.37	16.7	0.83	-0.26	1.01	1
NM3_uhed@03	1.76	10.62	0.23	8.9	0.29	6.04	0.41	2
NM3_uhed@04	1.77	11.33	0.36	10.0	0.56	6.21	0.74	2
NM3_uhed@05	1.75	10.56	0.22	10.7	0.18	5.04	0.29	2
NM3_uhed@06	1.78	6.86	0.28	10.3	0.31	1.54	0.45	1
NM3_uhed@07	1.80	11.13	0.28	8.2	0.30	6.92	0.44	2
NM3_uhed@08	1.80	10.82	0.27	8.4	0.34	6.50	0.48	2
NM3_uhed@09	1.76	9.89	0.25	10.5	0.18	4.50	0.31	2
NM3_uhed@10	1.76	6.69	0.41	14.8	0.70	-0.89	0.91	1
NM3_uhed@11	1.72	8.32	0.27	12.6	0.27	1.84	0.41	1 and 2
NM3_uhed@12	1.79	9.48	0.27	8.2	0.24	5.28	0.38	2
NM3_uhed@13	1.79	10.20	0.26	7.5	0.25	6.34	0.38	2
NM3_uhed@14	1.76	10.56	0.29	7.6	0.21	6.67	0.35	2
NM3_tad@1	2.43	1.68	0.28	3.3	0.60	-0.01	0.75	1
NM3_tad@03	2.33	1.12	0.36	2.5	0.48	-0.16	0.66	1
NM3_tad@05	2.38	2.60	0.25	5.0	0.16	0.03	0.29	1
NM3_tad@06	2.33	2.24	0.29	4.0	0.25	0.19	0.40	1
NM3_tad@07	2.28	1.69	0.23	4.0	0.29	-0.38	0.41	1
NM3_tad@08	2.29	1.30	0.32	3.0	0.34	-0.27	0.50	1
NM3_tad@09	2.31	2.27	0.25	4.2	0.39	0.10	0.51	1
NM3_tad@10	2.27	3.12	0.23	5.7	0.24	0.21	0.35	1
NM3_tad@11	2.23	1.52	0.31	2.4	0.40	0.31	0.56	1
NM3_tad@12	2.26	2.09	0.27	4.3	0.28	-0.14	0.41	1
NM3_tad@13	2.29	3.06	0.23	5.6	0.24	0.19	0.35	1
NM3_tad@14	2.26	2.15	0.23	3.4	0.29	0.37	0.40	1
NM3_tad@15	2.28	2.44	0.26	4.2	0.34	0.27	0.47	1
NM3_tad@16	2.04	2.12	0.35	3.6	0.74	0.26	0.91	1
NM3_tad@17	2.23	2.19	0.33	3.9	0.50	0.21	0.66	1
NM3_tad@18	2.15	3.95	0.28	7.3	0.35	0.21	0.49	1
NM3_tad@19	2.19	1.80	0.33	4.0	0.45	-0.24	0.61	1
NM3_tad@20	2.08	1.79	0.44	3.2	0.82	0.12	1.04	1
NM1_stomach@1	2.21	7.93	0.28	15.4	0.46	0.03	0.60	1
NM1_stomach@02	2.15	5.83	0.50	11.6	0.66	-0.10	0.91	1
NM1_stomach@03	2.21	7.49	0.42	15.4	0.65	-0.44	0.86	1
NM1_stomach@04	1.98	8.36	0.27	17.2	0.55	-0.45	0.68	1
NM1_stomach@05	2.19	4.89	0.24	9.9	0.39	-0.19	0.51	1
NM1_stomach@08	2.21	5.99	0.26	12.7	0.34	-0.52	0.47	1
NM1_stomach@09	2.15	5.72	0.47	12.6	0.74	-0.73	0.98	1
NM1_stomach@10	2.20	6.57	0.26	13.8	0.23	-0.54	0.36	1
NM1_stomach@11	2.17	8.31	0.24	15.8	0.38	0.20	0.50	1
NM1_stomach@12	2.18	7.00	0.26	14.4	0.32	-0.38	0.45	1

NM1_stomach@14	2.17	7.19	0.31	14.3	0.60	-0.15	0.76	1
NM1_stomach@17	2.13	6.29	0.24	11.8	0.39	0.23	0.51	1
NM1_uhed@1	2.28	4.95	0.25	7.6	0.26	1.03	0.39	2
NM1_uhed@02	2.25	7.56	0.32	15.8	0.48	-0.54	0.64	1
NM1_uhed@03	2.22	2.63	0.30	5.7	0.67	-0.29	0.82	1
NM1_uhed@04	2.24	7.67	0.24	14.3	0.31	0.31	0.43	1
NM1_uhed@06	2.26	8.54	0.28	15.8	0.47	0.45	0.61	1
NM1_uhed@08	2.22	7.15	0.30	15.5	0.26	-0.78	0.41	1
NM1_uhed@09	2.22	4.57	0.22	10.0	0.33	-0.55	0.44	1
NM1_uhed@10	2.26	10.15	0.29	7.7	0.24	6.18	0.38	2
NM1_uhed@11	2.27	10.92	0.26	8.0	0.27	6.81	0.39	2
NM1_uhed@12	2.23	2.55	0.26	5.8	0.43	-0.42	0.56	1
NM1_uhed@13	2.27	8.10	0.20	5.8	0.25	5.09	0.35	2
NM1_uhed@14	2.26	10.69	0.23	8.2	0.27	6.49	0.38	2
NM1_uhed@15	2.21	9.70	0.29	8.7	0.31	5.25	0.46	2
NM1_uhed@17	2.22	7.70	0.27	16.7	0.39	-0.86	0.53	1
NM1_uhed@18	2.22	7.70	0.22	14.6	0.50	0.20	0.60	1
NM4_posbnew@03	2.27	5.05	0.35	10.9	0.45	-0.53	0.63	1
NM4_posbnew@04	2.32	4.99	0.31	10.1	0.50	-0.18	0.65	1
NM4_posbnew@05	2.20	5.21	0.31	10.9	0.41	-0.37	0.57	1
NM4_posbnew@06	2.33	4.46	0.32	9.5	0.30	-0.43	0.46	1
NM4_posbnew@07	2.35	5.45	0.27	11.9	0.39	-0.67	0.52	1
NM4_posbnew@08	2.34	6.13	0.41	14.0	1.04	-1.06	1.24	1
NM4_posbnew@09	2.30	3.86	0.29	8.7	0.32	-0.60	0.46	1
NM4_posbnew@10	1.79	6.89	0.44	14.2	0.78	-0.41	0.99	1
NM4_posbnew@11	2.28	4.95	0.21	10.1	0.23	-0.25	0.33	1
NM4_posbnew@12	2.27	4.38	0.42	10.1	0.77	-0.81	0.98	1
NM4_posbnew@13	2.21	4.57	0.22	10.8	0.39	-0.98	0.50	1
NM4_posbnew@14	2.29	3.31	0.22	7.5	0.22	-0.56	0.33	1
NM4_posbnew@16	1.33	7.24	0.36	14.6	0.83	-0.25	1.01	1
NM4_posbnew@17	2.19	5.59	0.38	12.1	0.39	-0.64	0.58	1
NM4_posbnew@18	2.17	6.40	0.34	12.7	0.75	-0.14	0.92	1
NM4_posbnew@20	2.25	4.29	0.27	9.5	0.34	-0.60	0.48	1
NM4_posbeugbig@1	2.23	10.56	0.31	7.9	0.25	6.50	0.41	2
NM4_posbeusmall@1	2.23	7.30	0.30	8.9	0.22	2.71	0.36	2
NM4_dognew@1	2.25	5.99	0.33	12.6	0.22	-0.46	0.39	1
NM4_dognew@02	2.26	6.62	0.24	14.1	0.38	-0.63	0.51	1
NM4_dognew@03	2.25	8.08	0.21	16.7	0.31	-0.50	0.41	1
NM4_dognew@04	2.19	3.31	0.44	8.3	0.60	-0.96	0.82	1
NM4_dognew@05	2.39	6.47	0.32	14.1	0.58	-0.79	0.74	1
NM4_dognew@06	2.26	7.77	0.20	16.7	0.33	-0.77	0.43	1
NM4_dognew@07	2.35	5.95	0.52	13.5	1.04	-0.97	1.30	1
NM4_dognew@08	2.45	7.17	0.17	14.7	0.21	-0.38	0.30	1
NM4_dognew@09	2.41	5.57	0.23	12.2	0.26	-0.69	0.38	1
NM4_dognew@10	2.39	6.57	0.23	14.3	0.32	-0.76	0.43	1
NM4_dognew@11	2.39	6.58	0.27	13.5	0.56	-0.34	0.69	1
NM4_dognew@12	2.41	5.32	0.33	11.3	0.32	-0.50	0.49	1
NM4_dognew@13	2.43	5.19	0.19	11.4	0.24	-0.66	0.33	1

NM4_dognew@14	2.39	5.92	0.22	12.2	0.21	-0.32	0.32	1
NM4_dognew@15	2.34	7.82	0.38	15.6	0.39	-0.20	0.57	1
NM4_dognew@16	2.33	7.39	0.21	16.3	0.50	-0.96	0.60	1
NM4_dognew@17	2.35	7.34	0.27	15.1	0.35	-0.43	0.48	1
NM4_dognew@18	2.34	5.99	0.44	13.8	0.50	-1.10	0.72	1
NM4_dognew@19	2.32	8.42	0.32	17.5	0.63	-0.58	0.79	1
NM4_dognew@20	2.38	5.45	0.26	10.8	0.24	-0.10	0.37	1
NM4_dognew@21	1.78	7.20	0.41	16.1	0.96	-1.04	1.16	1
NM4_dognew@22	2.31	6.95	0.23	15.5	0.50	-1.00	0.62	1
NM4_dognew@23	2.33	7.28	0.30	14.6	0.54	-0.23	0.69	1
NM4_dognew@24	2.35	6.78	0.29	14.5	0.24	-0.64	0.39	1
NM4_dognew@25	2.26	8.14	0.35	16.6	0.44	-0.37	0.61	1
NM4_dognew@26	2.34	5.51	0.29	11.2	0.86	-0.22	1.01	1
NM4_dognew@27	2.29	6.14	0.28	11.3	0.51	0.33	0.65	1
NM4_dognew@28	2.35	6.49	0.26	13.4	0.47	-0.38	0.60	1
NM4_dognew@29	2.31	5.57	0.26	11.6	0.31	-0.39	0.44	1
NM4_posanew@08	2.16	5.21	0.29	9.7	0.38	0.24	0.53	1
NM4_posanew@10	2.04	6.41	0.46	11.1	0.75	0.69	0.97	1

*Pyrite type: see the text for full description of pyrite morphology. Briefly, type 1 pyrite are irregular aggregates of pyrite crystals. These aggregates can range from 10 µm to 500 µm in size and are composed of anhedral to euhedral (cubic) pyrite crystals. Type 2 pyrite are euhedral single crystals that can range from 10 µm up to 100 µm in size. They overgrow type 1 pyrite clusters

Table S5 SIMS analysis of in-house pyrite standard

	Primary beam Ip (nA)	Measured ^{32}S (10^8 cps)	^{32}S Relative standard error (‰)	Measured $^{33}S/^{32}S$	^{33}R correction factor	$\delta^{33}S_{(corr, V\text{-}CDT)}$ (‰)	$\delta^{33}S$ 1SE (‰)	Measured $^{34}S/^{32}S$	^{34}R correction factor	$\delta^{34}S_{(corr, V\text{-}CDT)}$ (‰)	$\delta^{34}S$ 1SE (‰)
NM_stdtest@11	1.44	2.299	2.014	0.007885	0.9991	0.13	0.13	0.04421	0.9992	0.07	0.30
NM_stdtest@12	1.43	2.283	2.254	0.007888	0.9988	0.13	0.17	0.04419	0.9995	0.07	0.28
NM_stdtest@14	1.12	1.675	2.477	0.007883	0.9993	0.13	0.30	0.04420	0.9994	0.07	0.26
NM_stdtest@15	1.11	1.664	2.755	0.007888	0.9987	0.13	0.25	0.04418	0.9998	0.07	0.19
NM_stdtest@16	1.10	1.638	2.733	0.007887	0.9990	0.13	0.23	0.04417	0.9999	0.07	0.18
NM_stdtest@17	1.10	1.633	2.490	0.007887	0.9989	0.13	0.25	0.04420	0.9994	0.07	0.20
NM_stdtest@18	1.09	1.631	2.464	0.007886	0.9990	0.13	0.26	0.04416	1.0001	0.07	0.28
NM_stdtest@19	1.09	1.632	2.503	0.007884	0.9993	0.13	0.23	0.04416	1.0001	0.07	0.18
NM_stdtest@20	1.09	1.637	2.553	0.007886	0.9991	0.13	0.21	0.04418	0.9997	0.07	0.25
NM_stdtest@21	1.09	1.635	2.419	0.007884	0.9993	0.13	0.29	0.04418	0.9998	0.07	0.22
NM_stdtest@22	1.83	2.743	3.737	0.007886	0.9990	0.13	0.11	0.04416	1.0002	0.07	0.18
NM_stdtest@24	1.84	2.726	3.638	0.007888	0.9988	0.13	0.14	0.04417	1.0000	0.07	0.16
NM_stdtest@25	1.84	2.711	3.541	0.007884	0.9993	0.13	0.23	0.04415	1.0003	0.07	0.20
NM_stdtest@26	1.55	2.363	3.527	0.007880	0.9998	0.13	0.24	0.04416	1.0003	0.07	0.18
NM_stdtest@27	1.55	2.366	3.609	0.007885	0.9991	0.13	0.21	0.04416	1.0002	0.07	0.16
NM_stdtest@28	1.55	2.373	3.727	0.007886	0.9991	0.13	0.15	0.04415	1.0005	0.07	0.16
NM_stdtest@29	1.55	2.407	3.280	0.007880	0.9998	0.13	0.13	0.04414	1.0006	0.07	0.18
NM_stdtest@30	1.40	2.170	3.499	0.007881	0.9997	0.13	0.18	0.04415	1.0004	0.07	0.13
NM_stdtest@31	1.39	2.157	3.590	0.007884	0.9993	0.13	0.17	0.04415	1.0004	0.07	0.18
NM_stdtest@32	1.38	2.149	3.857	0.007882	0.9995	0.13	0.26	0.04413	1.0008	0.07	0.16
NM_stdtest@33	1.37	2.106	3.475	0.007885	0.9991	0.13	0.26	0.04416	1.0002	0.07	0.16
NM_stdtest@35	1.08	1.649	2.561	0.007887	0.9989	0.13	0.19	0.04418	0.9998	0.07	0.16
NM_stdtest@36	1.08	1.659	2.596	0.007887	0.9988	0.13	0.25	0.04418	0.9998	0.07	0.19
NM_stdtest@37	1.08	1.658	3.381	0.007884	0.9992	0.13	0.19	0.04416	1.0001	0.07	0.13
NM_stdtest@38	1.07	1.632	3.079	0.007885	0.9992	0.13	0.17	0.04417	1.0000	0.07	0.15
NM_stdtest@39	1.49	2.350	2.339	0.007890	0.9985	0.13	0.23	0.04419	0.9995	0.07	0.28
NM_stdtest@40	1.50	2.357	2.827	0.007888	0.9988	0.13	0.19	0.04417	0.9999	0.07	0.22
NM_stdtest@41	1.50	2.354	2.867	0.007889	0.9986	0.13	0.28	0.04417	1.0000	0.07	0.19
NM_stdtest@42	1.50	2.361	3.201	0.007887	0.9989	0.13	0.17	0.04417	1.0000	0.07	0.22
NM_stdtest@43	1.32	2.099	2.553	0.007882	0.9995	0.13	0.15	0.04417	0.9999	0.07	0.14
NM_stdtest@44	1.34	2.117	3.273	0.007883	0.9994	0.13	0.28	0.04416	1.0003	0.07	0.21
NM_stdtest@46	1.31	2.055	3.148	0.007885	0.9992	0.13	0.26	0.04416	1.0003	0.07	0.20
NM_stdtest@47	1.30	2.064	3.247	0.007881	0.9996	0.13	0.15	0.04415	1.0003	0.07	0.18
NM_stdtest@48	1.30	2.051	3.276	0.007884	0.9993	0.13	0.15	0.04414	1.0006	0.07	0.18
NM_stdtest@49	1.30	2.050	3.087	0.007886	0.9990	0.13	0.24	0.04415	1.0003	0.07	0.17

Table S6 SIMS analysis of Balmat and in-house pyrite standard

	Primary beam Ip (nA)	Measured ^{32}S (x 10^8 cps)	^{32}S Relative standard error (‰)	Measured $^{33}S/^{32}S$	^{33}S Relative standard error (‰)	^{33}R correction factor	Measured $^{34}S/^{32}S$	^{34}S Relative standard error (‰)	^{34}R correction factor
CJ blmt_x-436y-1445	2.570	2.448	0.939	0.0079468	0.267	0.9989	0.044901	0.185	0.9984
CJ blmt_x-434y-1547	0.829	2.481	1.140	0.0079532	0.231	0.9981	0.044901	0.241	0.9984
CJ blmt_x-157y-1734	0.960	2.213	0.529	0.0079533	0.152	0.9981	0.044898	0.095	0.9985
CJ blmt_x-187y-1756	0.955	2.252	1.279	0.0079596	0.181	0.9973	0.044952	0.130	0.9973
CJ blmt_x-106y-1709	0.975	2.219	1.937	0.0079501	0.149	0.9985	0.044869	0.137	0.9991
CJ blmt_x-20y-1686	0.990	2.227	1.062	0.0079533	0.189	0.9981	0.044888	0.114	0.9987
CJ blmt_x27y-1513	0.994	2.278	2.014	0.0079554	0.214	0.9978	0.044884	0.177	0.9988
CJ blmt_x-123y-1581	0.963	2.295	1.578	0.0079530	0.266	0.9981	0.044911	0.125	0.9982
CJ blmt_x-211y-1652	0.941	2.288	1.237	0.0079492	0.237	0.9986	0.044863	0.118	0.9993
CJ blmt_x-418y-1524	0.863	2.427	0.675	0.0079565	0.188	0.9977	0.044929	0.126	0.9978
CJ4705std_x-8788y1723	0.980	2.257	2.584	0.0078831	0.163	0.9980	0.044219	0.187	0.9986
CJ4705std_x-8774y1742	0.983	2.250	2.347	0.0078925	0.163	0.9980	0.044194	0.154	0.9986
CJ4705std_x-8818y1719	0.986	2.250	2.764	0.0079002	0.227	0.9980	0.044303	0.199	0.9986
CJ4705std_x-8810y1733	0.988	2.265	2.587	0.0078975	0.150	0.9980	0.044281	0.195	0.9986
CJ4705std_x-8853y1691	0.987	2.259	2.521	0.0078973	0.148	0.9980	0.044261	0.187	0.9986
CJ4705std_x-8966y1684	0.989	2.251	2.547	0.0078968	0.173	0.9980	0.044236	0.206	0.9986
CJ4705std_x-9024y1688	0.989	2.249	2.652	0.0078939	0.103	0.9980	0.044232	0.209	0.9986
CJ4705std_x-8774y1764	0.980	2.236	1.745	0.0078919	0.213	0.9980	0.044201	0.180	0.9986
CJ4705std_x-8799y1753	0.981	2.261	2.409	0.0079010	0.170	0.9980	0.044281	0.166	0.9986
CJ4705std_x-8860y1742	0.983	2.252	2.267	0.0078935	0.213	0.9980	0.044198	0.196	0.9986
CJ4705std_x-8862y1808	0.985	2.265	2.959	0.0078932	0.206	0.9980	0.044214	0.229	0.9986
CJ4705std_x-8980y1775	0.982	2.260	2.424	0.0078906	0.156	0.9980	0.044177	0.182	0.9986
CJ blmt_x-365y-1655	2.435	2.337	0.865	0.0079509	0.179	0.9984	0.044840	0.132	0.9998
CJ blmt_x-238y-1752	0.796	2.192	2.457	0.0079540	0.189	0.9980	0.044866	0.143	0.9992
CJ blmt_x-102y-1740*	0.981	2.174	0.405	0.0079593	0.253	0.9974	0.044926	0.199	0.9979
CJ blmt_x20y-1695	0.882	2.152	1.659	0.0079565	0.207	0.9977	0.044909	0.201	0.9983
CJ blmt_x-167y-1645	0.845	2.177	1.413	0.0079621	0.121	0.9970	0.044952	0.130	0.9973
CJ blmt_x-353y-1430	0.797	2.381	0.646	0.0079522	0.206	0.9982	0.044873	0.138	0.9991
CJ blmt_x-332y-1378	0.802	2.395	1.384	0.0079508	0.207	0.9984	0.044805	0.154	1.0006
CJ blmt_x96y-1613	0.891	2.077	1.502	0.0079560	0.150	0.9978	0.044883	0.156	0.9988
CJ blmt_x32y-1556	0.882	2.218	2.308	0.0079597	0.212	0.9973	0.044968	0.142	0.9970
CJ blmt_x-226y-1445	0.825	2.371	0.260	0.0079555	0.160	0.9978	0.044887	0.134	0.9987

*The known $\delta^{33}S_{V-CDT}$ and $\delta^{34}S_{V-CDT}$ values of the Balmat standard are 7.7‰ and 15.1‰, respectively.

References

Crowe, DE, Vaughan RG (1996) Characterization and use of isotopically homogeneous standards for in situ laser microprobe analysis of 34S/32S ratios *American Mineralogist* **81**, 187–193.

Ding T, Valkiers S, Kipphardt H, De Bievre P, Taylor PDP, Gonfiantini R, Krouse R (2001) Calibrated sulfur isotope abundance ratios of three IAEA sulfur isotope reference materials and V-CDT with a reassessment of the atomic weight of sulfur. *Geochimica et Cosmochimica Acta* **65,** 2433–2437.

Ushikubo T, Williford KH, Farquhar J, Johnston DT, Van Kranendonk MJ, Valley JW (2014) Development of in situ sulfur four-isotope analysis with multiple Faraday cup detectors by SIMS and application to pyrite grains in a Paleoproterozoic glaciogenic sandstone. *Chemical Geology* **383**, 86–99.
